# Heterogeneous Catalytic Conversion of Sugars Into 2,5-Furandicarboxylic Acid

**DOI:** 10.3389/fchem.2020.00659

**Published:** 2020-07-31

**Authors:** Athukoralalage Don K. Deshan, Luqman Atanda, Lalehvash Moghaddam, Darryn W. Rackemann, Jorge Beltramini, William O. S. Doherty

**Affiliations:** ^1^Centre for Agriculture and the Bioeconomy, Queensland University of Technology, Brisbane, QLD, Australia; ^2^IROAST, Department of Chemistry, Faculty of Advanced Science and Technology, Kumamoto University, Kumamoto, Japan

**Keywords:** 2,5-furandicarboxylic acid (FDCA), sugars, FDCA derivatives, heterogeneous catalysis, catalysis, 5-(hydroxymethyl)furfural (HMF)

## Abstract

Achieving the goal of living in a sustainable and greener society, will need the chemical industry to move away from petroleum-based refineries to bio-refineries. This aim can be achieved by using biomass as the feedstock to produce platform chemicals. A platform chemical, 2,5-furandicarboxylic acid (FDCA) has gained much attention in recent years because of its chemical attributes as it can be used to produce green polymers such polyethylene 2,5-furandicarboxylate (PEF) that is an alternative to polyethylene terephthalate (PET) produced from fossil fuel. Typically, 5-(hydroxymethyl)furfural (HMF), an intermediate product of the acid dehydration of sugars, can be used as a precursor for the production of FDCA, and this transformation reaction has been extensively studied using both homogeneous and heterogeneous catalysts in different reaction media such as basic, neutral, and acidic media. In addition to the use of catalysts, conversion of HMF to FDCA occurs in the presence of oxidants such as air, O_2_, H_2_O_2_, and *t*-BuOOH. Among them, O_2_ has been the preferred oxidant due to its low cost and availability. However, due to the low stability of HMF and high processing cost to convert HMF to FDCA, researchers are studying the direct conversion of carbohydrates and biomass using both a single- and multi-phase approach for FDCA production. As there are issues arising from FDCA purification, much attention is now being paid to produce FDCA derivatives such as 2, 5-furandicarboxylic acid dimethyl ester (FDCDM) to circumvent these problems. Despite these technical barriers, what is pivotal to achieve in a cost-effective manner high yields of FDCA and derivatives, is the design of highly efficient, stable, and selective multi-functional catalysts. In this review, we summarize in detail the advances in the reaction chemistry, catalysts, and operating conditions for FDCA production from sugars and carbohydrates.

## Introduction

The global economy depends on energy, chemicals, materials, and water for sustainability. The chemical industry is one of the dominant industries as chemicals are used in a variety of applications including in large scale production of fuels and fuel additives, polymeric materials and chemical intermediates for example, as hosting matrix for nanocomposites, nanocatalysts, anticorrosion coatings, membrane for energy unit, and so on (Sheldon, 2014; Bulushev and Ross, 2018; Qian et al., 2018; Gu et al., 2019; He et al., 2019; Jiang et al., 2019; Li et al., 2019; Ma et al., 2019; Shi et al., 2019; Yao et al., 2019; Yuan et al., 2019; Zhang et al., 2019; Zhao et al., 2019; Zheng et al., 2019, 2020). However, most chemical industries depend on fossil-derived feedstocks and so are not sustainable and do irreparable damage to the environment by the massive carbon emissions they generate. At present, a global temperature rise of 1.5°C by 2050 is a best-case-scenario that will require a rapid reduction in carbon emissions, which cannot be achieved by improvements in renewable energy and energy efficiency alone. For a cleaner and healthier planet, nations must transition from “take-make-waste” *linear economies* that are extractive, resource-intensive, and wasteful to *circular economies*, in which materials and products are produced, used, and re-used in ways that maximize value and minimize negative impact.

Polymeric materials, such as polyamides and polyesters like polyethylene terephthalate (PET), derived from fossil fuels, are important platform materials utilized today for the production of a wide range of products (Lancefield et al., 2018). However, similar materials can be produced from 2,5-furandicarboxylic acid (FDCA), a biobased chemical monomer. For example, FDCA can used to produce polyethylene 2,5-furandicarboxylate (PEF), which has similar properties as PET. FDCA can be synthesized directly from renewable biomass/carbohydrate and hence reduce our dependent on fossil fuels. The conversion of simple sugars to FDCA is a more feasible approach compared to the conversion of lignocellulosic biomass because the latter is recalcitrant and multiple reaction steps are required to unlock the sugars, i.e., glucose from the cell wall matrix. The conversion of sugars to FDCA involves dehydration to 5-(hydroxymethyl)furfural (HMF) as the first step followed by its oxidation to FDCA. This process can be achieved by non-catalytic, catalytic (chemical or biological) or electrochemical conversion methods. Nonetheless, the catalytic processes have been used to produce the highest yields of FDCA with high conversion rates (Sajid et al., 2018). That said, there remains the challenge of separation and purification of HMF from the reaction media, and using high HMF loading. To overcome this, chemical catalytic conversion of sugars into FDCA without removing HMF from the reaction medium is being explored. Processes that are amenable to directly convert sugars to FDCA has several advantages including reduced CO_2_ emissions, reduced energy consumption, and lower cost since sugars are a relatively cheaper feedstock than HMF (Menegazzo et al., 2018). Therefore, FDCA derived from sugars can be obtained in an innovative way using a one-pot synthesis method. However, this method is yet to be perfected. HMF can also be synthesized by microwave activation of sugars by using homogeneous catalysis, heterogeneous catalysis, and non-catalytic methods. However, environmental, toxicity, and operational issues are the major drawbacks of using homogeneous catalysts. Heterogeneous catalysts such as ZrO_2_ with sulfates (${\text{SO}}_{4}^{2-}$) and molybdene oxides or tungsten oxides have resulted 83 and 82% of increased HMF yield at microwave heating at 150°C for 5 min (Wang J. et al., 2015). Moreover, sulphated polyvinyl alcohol as solvent has resulted in 85% of HMF yield from fructose by microwave heating at 130°C for 2 min (Pawar and Lali, 2016). Microwave assisted reaction of fructose in 1-butyl-3-methylimidazole chloride ([BMIM]Cl) ionic liquid was reported to give 98% of HMF at 155°C after 1 min of heating time (Li et al., 2011). 5-(chloromethyl) furfural (CMF), also produced from sugars, can be converted to FDCA, as it has similar chemical functionality as HMF. CMF has the distinction that it can be produced in high yield directly from biomass and is more easily separated due to its hydrophobic character (Mascal, 2015). Chundury and Szmant (1981) reported the use of nitric acid to improve the oxidation of CMF to FDCA. Following this work, Brasholz et al. (2011) were able to obtain a 59% yield of FDCA. Nonetheless, the conversion of CMF to FDCA is still an emerging field and needs extensive studies before its potential for large scale production of FDCA is realized.

The most extensively studied pathways for the oxidation of HMF to FDCA use either homogeneous or heterogeneous catalysis. Homogeneous catalysts have numerous drawbacks including low FDCA yield, high by-product formation as well as difficulties in catalyst separation and product purification. A recent review reports on advances in the catalytic synthesis of FDCA, wherein FDCA is produced from the oxidation of HMF, or directly from sugars by a one-step reaction process (Zhang and Deng, 2015). The excellent review by Sajid et al. (2018) compares the use of noble metal catalysts for FDCA production, but these types of catalysts are of high cost, compared to their transition metal counterparts, and details of the status of FDCA manufacture was presented. The present review complements recent reviews, by providing additional information on current and past industrial processes for FDCA manufacture, FDCA production via different oxidants such as oxygen, hydrogen peroxide (H_2_O_2_), and other oxidants and further discusses the effect of noble metals, non-noble metals, and metal free catalysts with different supports. Moreover, the effect of neutral (i.e., base-free) and basic media and associated kinetics of these processes are also discussed with regards to the oxidation of HMF to FDCA. This review describes both the single-phase and multiphase reactions to directly convert sugars to FDCA, highlighting ways to effectively recover FDCA and strategies to minimize impurity contamination of FDCA. The review concludes by highlighting future areas of research to address issues around the competitiveness of FDCA production as a commodity chemical.

## Importance of Fdca and Derivatives

**Scheme 1:**
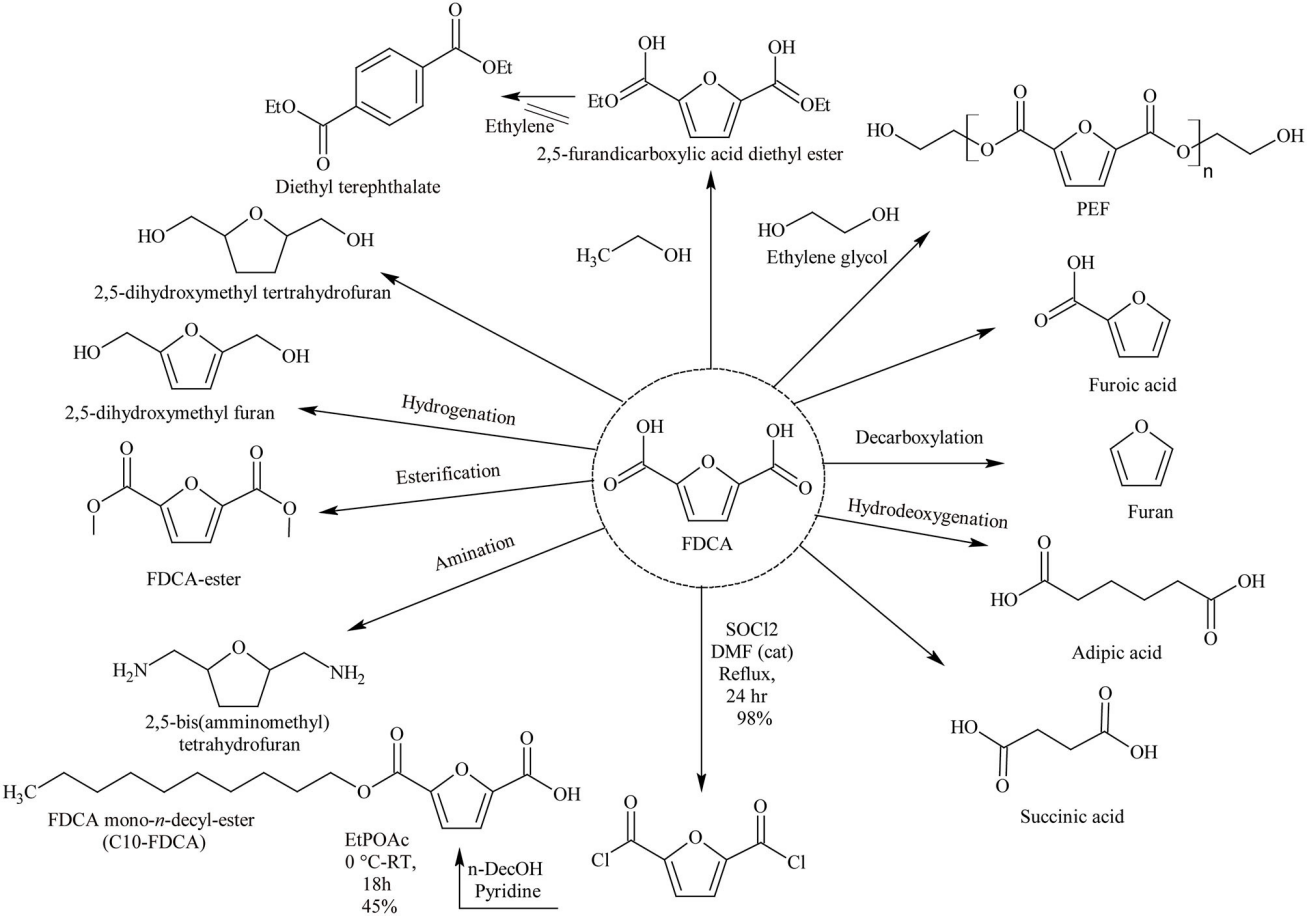
Chemicals derived from FDCA. Reproduced with permission from Pal and Saravanamurugan (2019) copyright (2019) Wiley online library.

FDCA is a furan chemical and so has a cyclic structure with di-acidic side chains (Auvergne et al., 2013). FDCA is considered as a platform chemical because it can be readily converted to various products of higher value (Takkellapati et al., 2018). However, the term platform chemicals are used frequently in literature with different emphasis. According to the US Department of Energy (DOE) report, “molecules with multiple functional groups that possess the potential to be transformed into new families of useful molecules” are considered as platform chemicals (Bozell and Petersen, 2010). José-Vitor Bomtempo and team has defined six parameters for a chemical to be considered a platform chemical. These include: the chemical has to be an intermediate; with a flexible structure to produce various derivatives; cost-effective at the platform chemical level and derivatives level; create value in scale and scope economies; be structured in innovation ecosystems; and finally have developed mechanisms of governance (Bomtempo et al., 2017). DOE has listed FDCA among the top 12 value-added chemicals, which are considered as platform chemicals owing to their capacity to function as a precursor for the synthesis of a wide range of chemicals (Takkellapati et al., 2018). The versatility and range of derivatives from FDCA is illustrated in Scheme 1. For example, FDCA can be used to produce biochemicals like succinic acid, fungicides, isodecylfuran-2,5-dicarboxylate, and poly(ethylenedodecanedioate-2,5-furandicarboxylate) (Zhang and Deng, 2015). FDCA can also be used as a substitute for terephthalic acid, which is used to produce polyethylene terephthalate (PET) (Sajid et al., 2018). A solvent-less path has also been used to synthesize diethyl terephthalate from FDCA (Ogunjobi et al., 2019). The use of FDCA and its decyl ester to produce surfactants have been discussed by Van Es et al. (2013). FDCA can serve as a platform chemical for monomers like 2,5-furandicarboxylic dichloride (FDCDCl) to produce nylons by reacting with diamines or forming 2,5-bis(aminomethyl)tetrahydrofuran (Sajid et al., 2018). Other derivative applications of FDCA include corrosion inhibitors, pharmaceutical intermediates, cross-linking agents, and medicinal products (Kröger et al., 2000; Sajid et al., 2018).

## Past and Current Industrial FDCA Production

**Scheme 2:**
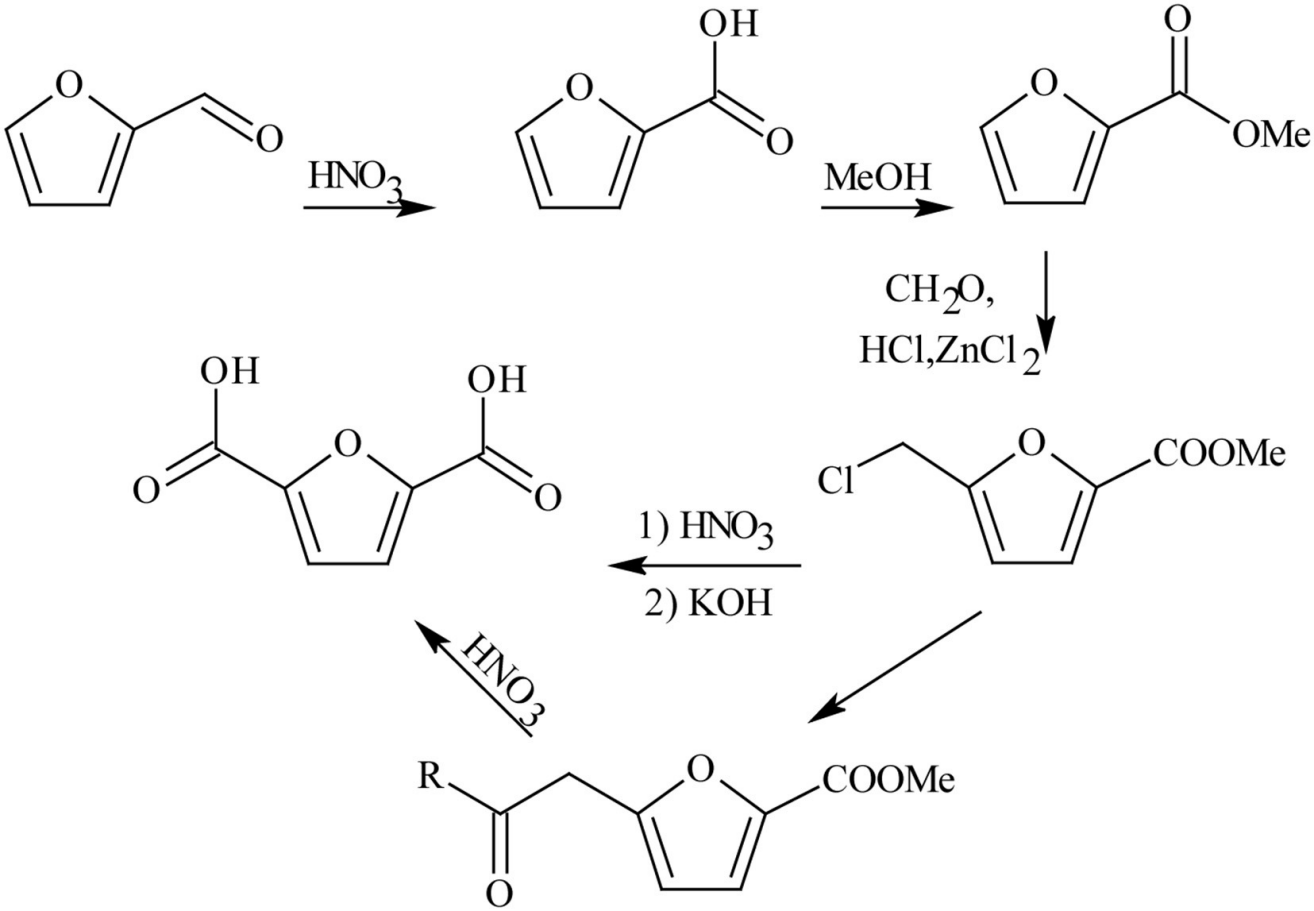
FDCA synthesis from furfural. Reproduced with permission from Gonis and Amstutz (1962) copyright (1962) American Chemical Society.

FDCA production started long time ago in 1876, where mucic acid (48% in aqueous solution) was reacted with hydrogen bromide (HBr). However, this method was not commercially viable due to high cost of material, elevated working temperature, and long reaction time (Rose et al., 2013), resulting in the search for a substitute to mucic acid as a reactant (Gonis and Amstutz, 1962). Furfural was explored as a replacement to produce FDCA, via a multi-step oxidation and esterification reaction pathways as shown in Scheme 2 (Gonis and Amstutz, 1962; Rose et al., 2013). Unfortunately, this is a low efficient pathway that resulted in poor FDCA yield and selectivity with many intermediate products. Contrastingly, carboxylation reaction pathway of furfural to FDCA has gained much attention because furfural can be readily oxidized to furoic acid under mild conditions (Taarning et al., 2008; Gupta et al., 2017a) and subsequent carboxylation can be achieved without any solvent or transition metal catalysts (Dick et al., 2017). Consequently, Zhang et al. (2018a) was able to demonstrate that 5-bromo-furoic acid, obtained by the bromination of furfural-derived furoic acid, can effectively undergo aqueous phase carbonylation to produce high yield (98%) of FDCA and facilely separated through a simple acidification of the reaction medium. Another route to synthesize FDCA and found to be most effective is the oxidation of HMF, a C6 based acid dehydration product of carbohydrates. It can be oxidized to FDCA using homogeneous and heterogeneous catalysts, biocatalysts, electrochemical oxidation, or even without any catalyst (Dijkman et al., 2014; Zhang and Deng, 2015; Nam et al., 2018; Xuan et al., 2018; Yan et al., 2018a). Table 1 compares the advantages and disadvantages of these types of catalytic methods wherein chemical catalytic methods (homogeneous and heterogeneous catalytic methods) show good productivity, with heterogeneous catalysis showing the most promising technology.

**Table 1:** Summary of the different HMF oxidation processes to FDCA.

**Entry**	**Homogeneous catalysts**	**Heterogeneous catalysts**	**Biocatalysts**	**Electrochemical oxidation**
1)	Difficult to separate	Facile separation	Possible facile separation	Facile separation
2)	Recycling deficiencies	Facile recyclability	Recycling deficiencies	Recycling deficiencies
3)	Low yields with mild conditions	Low yields with mild conditions	Mild conditions (ambient temperature and atmospheric pressure) are used	Mild conditions are required
4)	Oxidant is needed	Oxidant is needed	Oxidant may or may not be needed	External oxidant is not necessary
5)	Less environmentally friendly	When inorganic base used, process becomes less environmentally friendly	Green process	Green process

Currently, there is limited commercial production of FDCA, and only two larger scale developments are of importance. Avantium has demonstrated a pilot production of FDCA from HMF ethers based on the Avantium YXY^®^ process using sugars as the primary feedstock (Sajid et al., 2018). The other one is a continuous, integrated pilot plant for the aqueous oxidation of HMF with paired electrochemical oxidation developed by AVA Biochem to obtain and purify FDCA. In this case, HMF is sourced from the hydrothermal processing of sugars, although their long-term goal is to produce HMF from cheaper biomass sources.

**Scheme 3:**
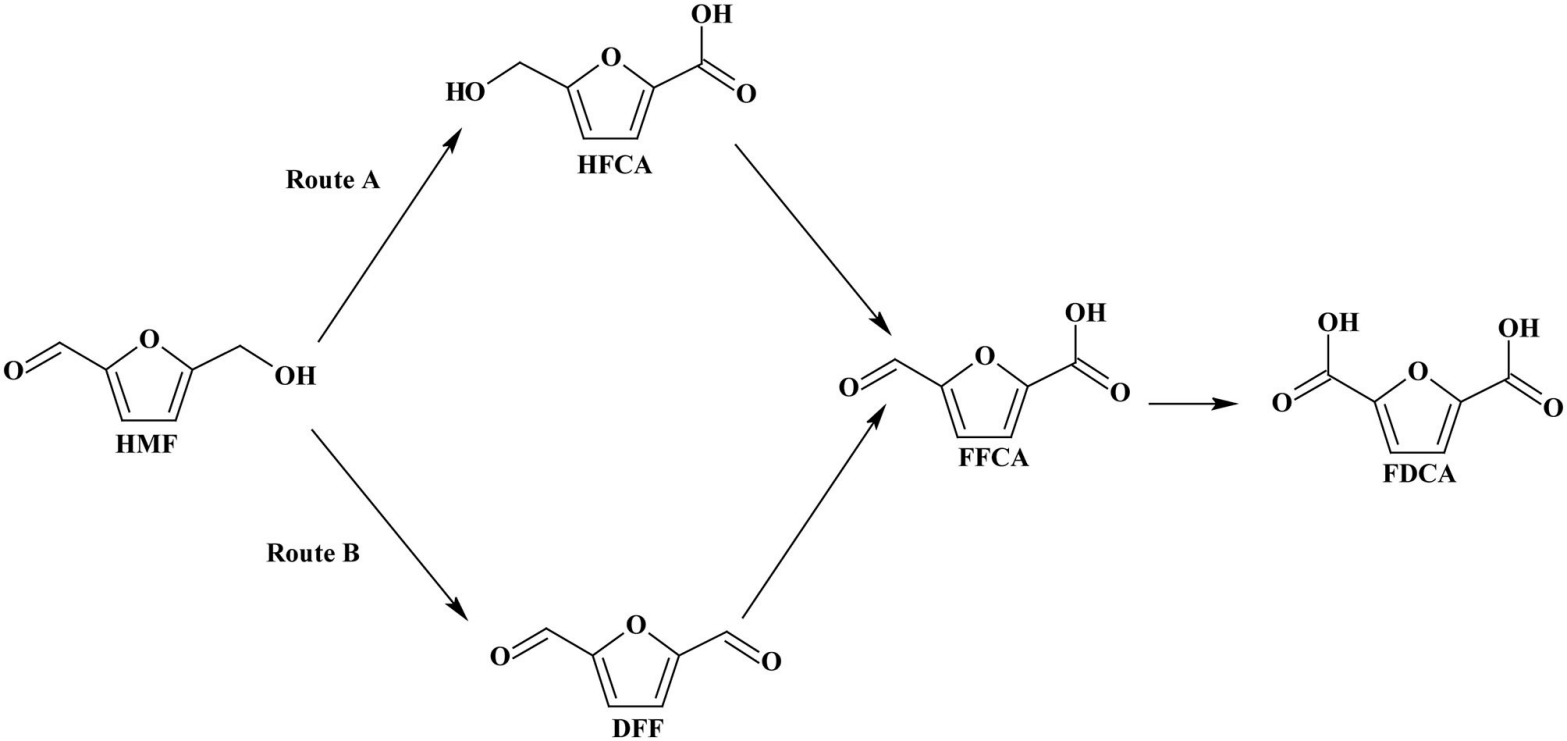
Reaction routes of HMF oxidation to FDCA. Reproduced with permission from Sajid et al. (2018) copyright (2018) Royal Society of Chemistry (RSC) publishing.

At the present time, there is no commercial process to produce FDCA from lignocellulosic biomass. The production of FDCA from biomass consists of multi-reaction steps, which complicates process design. In the first step, cellulose is separated from hemicellulose and lignin and in the second step hydrolyzed to glucose. The glucose is isomerized to fructose and the latter is dehydrated to HMF and in the final step via oxidation to FDCA (Tan et al., 2019). HMF oxidation to FDCA can occur by two reaction pathways as shown in Scheme 3. One reaction pathway involves oxidation of the aldehyde functional group of HMF to form the intermediate 5-hydroxymethyl-2-furancorboxylic acid (HFCA) or, via alcoholic group oxidation to produce 2,5-diformylfuran (DFF) as intermediate compound. Complete oxidation of these intermediates to FDCA then proceeds via a second intermediate product, 5-formyl-2-furancaboxylic acid (FFCA).

## Heterogeneous Catalytic Oxidation of HMF to FDCA

Production of FDCA from HMF using heterogeneous catalysts is more advantageous than the use of homogeneous catalysts because of higher FDCA yield, easier separation and purification of FDCA and lower by-product yields. As such, extensive studies have been conducted on the heterogeneous catalytic oxidation of HMF to FDCA over various oxidizing agents. Oxidizing agents play significant roles in HMF oxidation by facilitating the oxidization on the catalyst surface (Davis et al., 2012, 2014). Molecular oxygen (O_2_) or air, H_2_O_2_, and various other oxidants have been used in the oxidation of HMF to FDCA and are discussed in the following sections.

### Oxygen as an Oxidant

Among the various type of oxidants, O_2_ has been extensively studied due to its high oxidation ability, low cost, its availability, and being environmental friendly (Sajid et al., 2018). Due to the fact that O_2_ has a high activation energy, supported noble metals like gold (Au), palladium (Pd), platinum (Pt) ruthenium (Ru), and rhodium (Rh) have been extensively used as the main heterogeneous catalysts for the aerobic oxidation of HMF to FDCA. Apart from the noble metal heterogeneous catalysts, transition metal supported catalysts, and metal free heterogeneous catalysts have also been used in the aerobic oxidation process of HMF to FDCA.

#### Effect of Noble Metal Supported Heterogeneous Catalysts Over the Aerobic Oxidation of HMF to FDCA

Noble metals supported catalysts have been extensively used in aerobic oxidation of HMF to FDCA, due to their high catalytic activity, stability, and recyclability (Zhang and Deng, 2015). As shown in Scheme 3 route A, the reaction pathway of HMF oxidation involves aldehyde group oxidation to HFCA and hemiacetal intermediates. Apart from the type of noble metal, catalyst support, alloys of noble metals, and the reaction media are the other parameters which significantly affect the aerobic oxidation of HMF to FDCA. For noble metal heterogeneous catalysis, the catalyst support enhances the oxidation of HMF to FDCA as the material affects the activity of the catalyst (Sahu and Dhepe, 2014). Among the different support materials, carbon materials are broadly used as support because they are cheap, have desired properties, and are readily available. Davis et al. (2011) have discussed the effect of Pt, Pd, and Au noble metals on carbon support under identical reaction conditions of 6.9 bar O_2_ pressure for 6 h at 23°C and observed relatively low yield (8%) with Au/C catalyst. This was due to the difficulty of Au to activate the alcoholic side chain of HFCA before the latter was oxidized to FDCA. However, at a higher concentration of a base (e.g., NaOH) and a higher pressure of O_2_ the yield of FDCA increased, apparently due to the increase in the base concentration (Davis et al., 2011). Villa et al. (2013) have investigated the effect of activated carbon (AC) support over the Au, Pt, and Pd catalysts (of similar particle diameter in the range 2.9-3.9 nm) in the aerobic oxidation of HMF to FDCA. The HMF oxidation reaction was carried out at 3 bar O_2_ pressure at 60°C temperature in NaOH solvent and observed the highest yield of FDCA (80%) with Au/AC catalyst. However, when the catalysts were protected with polyvinyl alcohol (PVA) (Villa et al., 2013), whilst the highest FDCA yield was obtained with Au/AC, the FDCA yields with Pt/AC and Pd/AC were only 20 and 9%, respectively. It was found that the Pd and Pt were deactivated and the main product was HFCA rather than FDCA (Villa et al., 2013). Villa et al. (2013) went further and examined the effect of different carbon supports like, AC, carbon nanofibers (CNFs), carbon nanotubes (CNTs), and graphite (Graph). The FDCA yield of 80% with Au/AC, 6% with Au/CNFs, 39% with Au/CNTs, and 26% with Au/Graph (Villa et al., 2013). The highest yield of FDCA (80%) was observed with Au/AC due to its ability to oxidize both the steps of HMF to HFCA and HFCA to FDCA. It is likely therefore that the architecture and surface properties of the carbon plays vital role in the conversion process. It should be noted, however, that the residual K on the surface of AC had no noticeable effect on FDCA yield of Au/AC (Villa et al., 2013). Kerdi et al. (2015) elegant work on Ru-based catalysts clearly provided further evidence on the effect of surface properties on the oxidation of HMF to FDCA. It took the catalyst, Ru impregnated activated carbon (Ru_imp_/AC) 2 h at 100°C and 40 bar for total conversion of HMF, but a longer time of 4 h for the catalyst, prepared by cationic exchange of Ru on NaOCl oxidized activated carbon (Ru_ex_/AC_NaOCl_). This is because, the surface of the carbon-treated NaOCl introduced some of the oxygenated groups (e.g., carboxylic), some may have broken down during carbonization, and the residual oxygen functionality impacted negatively on the reaction rate for the formation of FDCA. Further evidence of the carbon architecture and surface properties on catalytic activity was demonstrated by Niu et al. (2014) who found that the use of reduced graphene oxide (RGO) gave a FDCA yield of 84% with Pt/RGO by oxidizing HMF via forming 5-hydroxymethyl-2-furancarboxylic acid (HMFCA) as an intermediate. Moreover, carbon supported Ru catalyst (5% Ru/C) was used in the microwave assisted HMF oxidation in air for the first time by Zhao et al. (2020b) and observed 84% FDCA yield after 22 h with the addition of strong base NaOH.

To improve catalyst recovery, reduce operating and catalyst costs, and recyclability, magnetic core-shell structured Fe_3_O_4_@C was prepared and used as a support for Pt, and exhibited a high yield (84%) of FDCA after 4 h at 90°C, in a Na_2_CO_3_ alkaline media at 1 bar O_2_ pressure (Zhang et al., 2016). The catalyst could be recycled at least three times without any decrease in its catalytic performance. Apart from carbon, the effect of CeO_2_ as a catalyst support was investigated by Kim et al. (2018) because of its Lewis acid and hydroxide ion sites. Kim et al.'s work with Au/CeO_2_ in the aerobic oxidation of low aqueous concentrations of HMF and its cyclic acetal derivative gave a yield of 90–95% FDCA (Kim et al., 2018). However, for a concentrated solution of 10 wt% under 2.0 equivalent Na_2_CO_3_ FDCA yield from HMF declined to 28%, whereas oxidation of 1,3-propanediol derived cyclic acetal of HMF (PD-HMF) gave a high yield of FDCA at 94%. More so, at higher concentrations of PD-HMF more than 10 wt%, FDCA yield stabilized above 90%. It was concluded that the reactive formyl group of HMF can be stabilized by acetalization with 1,3-propanediol to produce a stable intermediate cyclic HMF-acetal that overcomes the thermal instability of HMF which inherently limits selective oxidation to FDCA.

**Scheme 4:**
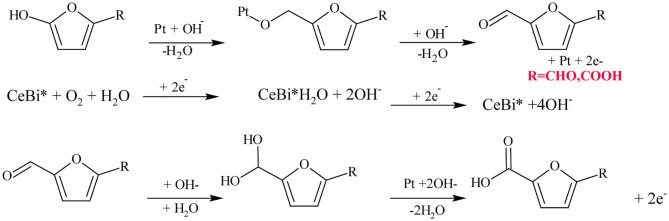
Reaction mechanism of aerobic oxidation of HMF to FDCA in alkaline media over Pt/Ce/Bi catalyst. Reproduced with permission from Miao et al. (2015) copyright (2015) Royal Society of Chemistry.

Miao et al. (2015) have investigated the catalytic effect of Pt/CeO_2_ by addition of Bi. The Pt/Ce_0_._8_Bi_0_._2_O_2−δ_ gave improved FDCA yield of 98% working under 10 bar of O_2_ pressure at 23°C in NaOH, while only Pt/CeO_2_ gave only 20% of FDCA yield. Moreover, Pt/Ce_0_._8_Bi_0_._2_O_2−δ_ catalyst showed excellent stability and reusability (around five runs with only a small reduction in FDCA yield). Considering the reaction mechanism of HMF oxidation by Pt/Ce_0_._8_Bi_0_._2_O_2−δ_ catalyst in alkaline media as shown in Scheme 4, Pt-alkoxides was formed as an intermediate because Pt substituted β-H with hydroxide ions (OH^−^) in the hydroxide groups of HMF. Then, oxygen vacancies and breakdown of transitional peroxides accelerated oxygen reduction in Bi-Ce complex, generating surface electrons that are continuously consumed, consequently safeguarding the catalytic efficiency even up to five times of recycling (Miao et al., 2015). Sahu and Dhepe (2014) have compared the catalytic ability of Pt catalysts supported on reducible oxides (TiO_2_ and CeO_2_) and non-reducible oxides (ZrO_2_, Al_2_O_3_, and C) over the oxidation of HMF to FDCA under 1 bar O_2_ pressure at 75°C in Na_2_CO_3_ media for 12 h (). It was observed that Pt catalysts supported on reducible oxides gave poor FDCA yield around 2–8% while, non-reducible oxides gave above 90% FDCA yield (Sahu and Dhepe, 2014). According to these results, it could be inferred that the low oxygen storage capacity of non-reducible oxides has translated to higher FDCA yield by preventing the oxidation of active metal sites. Zeolite is another support material which has been used in Au catalysts for the oxidation of HMF (Cai et al., 2013). When compared to other supports on Au catalyst such as Mg(OH)_2_, TiO_2_, CeO_2_, and ZSM-5; zeolite showed >99% FDCA yield at 60°C under 0.3 bar O_2_ pressure after 6 h. These results were explained on the basis of the stabilization of the dispersed Au particles, which were encapsulated inside the H-Y-zeolite super-cage to prevent further agglomeration (Cai et al., 2013). Au on hydrotalcite (HT) as a basic support was investigated by Gupta et al. and was compared to neutral supports such as Al_2_O_3_ and C, and the acidic support SiO_2_, in a base-free media (Haruta, 2002; Gupta et al., 2011). An improved catalytic activity of >99% FDCA selectivity with complete conversion of HMF was observed with Au/HT compared to Au/Al_2_O_3_, Au/C and Au/SiO_2_ in the aerobic oxidation of HMF to FDCA in aqueous media. It was concluded that the effectiveness of the basic HT support to selectively oxidize HMF to FDCA under a base-free environment is due to its basicity which has the capacity to form intermediate hemiacetals from HMF and FFCA, and the formation of metal alcoholate species via metal-hydride shift from the HMFCA intermediate. However, when MgO which has higher basicity was used as a support material for Au catalyst in HMF oxidation to FDCA a reduced catalytic activity was observed. Thus, although the basicity of support is important, generating metal active sites and metal particle aggregation play additive roles (Takagaki et al., 2010; Gupta et al., 2011). Importantly, Gupta et al. (2011) found out that the catalytic activity of Au/HT is still stable even after three reaction cycles maintaining FDCA yields >90%. In another approach, the catalytic aerobic oxidation of HMF over Pd nanoparticles supported on HT (*x*Pd/HT-*n*) was studied by Wang et al. (2016). They observed that Mg/Al molar ratio and Pd loadings have significant influence on the catalyst activity and selectivity to FDCA. 2%Pd/HT-5 catalyst exhibited the best catalytic efficiency which corresponds to 2% Pd loading and Mg/Al molar ratio of 5; achieving 91.9% FDCA yield at 99.4% HMF conversion after 3 h of the reaction. Its remarkable catalytic performance was attributed to the suitable basicity of the Mg–Al–CO_3_ and the abundant weak basic sites (OH^−^ groups) present on the surface of HT. This catalyst can be reused five times with good recyclability and catalytic stability.

Apart from the catalyst support, the alloying of noble metals as catalyst material has been evaluated for the aerobic oxidation of HMF to FDCA. The concept of alloying was considered because of the easiness of tuning the physical and chemical properties of the catalyst and their activity (Hameed et al., 2020). Pasini et al. (2011) have compared Au-Cu/TiO_2_ bimetal catalyst with Au/TiO_2_ and Cu/TiO_2_ monometallic catalysts and observed no FDCA formed with Cu/TiO_2_, 10% with Au/TiO_2_ and 30% with the alloy as shown in Table 2. Au-Cu/TiO_2_ bimetal catalyst also tested at various Au/Cu mole ratios, and the alloy of various composition showed improved catalytic activity compared to the monometallic catalysts. The reason for the high catalytic activity of the Au-Cu/TiO_2_ catalyst lies on the isolation of Au sites by alloying with Cu. As such, Au-Cu/TiO_2_ can be reused and recovered without significant leaching and agglomeration. A synergistic effect was observed for the bimetallic catalysts due to the derived benefit of improving the stability of the catalyst and resistance to poisoning (Pasini et al., 2011). Lolli et al. (2015) have compared the HMF oxidation with the Pd-Au/TiO_2_ nanoparticle bimetallic catalyst with Pd/TiO_2_ and Au/TiO_2_ monometallic counterparts and identified that FDCA yield could be increased from 9 and 13 to 85%, respectively with 100% HMF conversion at 70°C at 10 bar O_2_ pressure after 4 h by alloying Pd with Au in 1:6 ratio. Lolli et al. (2015) have discussed that after the formation of HMFCA as an intermediate in the formation of FDCA, the bimetallic catalyst guides the reaction pathway without any further oxidation because of the difficulties of Pd and its alloy to perform the hydroxymethyl group oxidation of HMFCA. Similarly, Xia et al. (2019) compared the catalytic effect of the Pd-Au bimetal catalyst on HT support and monometallic counterparts (Pd/HT and Au/HT) catalysts and up to 90% FDCA yield was observed by bimetallic Pd-Au/HT catalyst with the ratio of Au:Pd = 4 at 60°C temperature in 1 bar pressure after 6 h. However, Au/HT was unable to oxidize HMFCA to FDCA, due to the covering of active sites of Au by HMF and HMFCA (Ardemani et al., 2015). Improved catalytic performance was because the bimetal catalyst has a smaller particle size than the monometallic catalyst, and the synergistic effect between two metals guides the HMF oxidation to FDCA (Xia et al., 2019). On other works, Au-Pd/AC catalyst has also shown similar catalytic activity with an 8:2 molar ratio of Au with Pd providing the best performance (Villa et al., 2013). Even though, the monometallic Au/AC catalyst showed good FDCA selectivity of 80%, it was affected by catalyst deactivation due to the irreversible adsorption of by-products and intermediates and also due to the Au particle agglomeration. After reuse and tested on five cycles, Au-Pd/AC catalyst shows high stability and still achieved 99% FDCA yield. Verdeguer et al. (1993) have conducted work with Pt/C over the oxidation of HMF to FDCA and achieved 81% yield of FDCA under 1 bar O_2_ pressure at 25°C in 1.25 M NaOH. These researchers then incorporated Pb into the Pt/C catalyst and observed 99% FDCA yield. HFCA was the intermediate with Pb/Pt catalyst suggesting the aldehyde group oxidation as shown in Scheme 3 route A. From these results it was inferred that the FDCA yield of Pt/C was increased with the bimetallic catalysts as shown in Table 2. Also Rass et al. (2013) have investigated the carbon-supported bismuth (Bi) Pt catalyst (Pt-Bi/C) with Bi:Pt molar ratio of 0.2 for the oxidation of HMF under 40 bar air pressure in 2 equiv. Na_2_CO_3_ media and obtained the FDCA yield of over 99%, which was significantly higher than with Pt/C alone (69%). In this reaction, HFCA and FFCA observed as process intermediates and then oxidized to FDCA. For the Pt-Bi/C catalyst, oxidation of FFCA to FDCA is the rate-determining step. Incorporating Bi into the Pt/C catalyst increased the stability and catalyst life by removing the oxygen poisoning and Pt leaching likelihood. Apart from that, Gupta et al. (2017b) have studied the bimetallic alloy nanoparticles of M_0.9_-Pd_0.1_ where M= Ni, Co, or Cu supported on Mg(OH)_2_, which is *in situ* prepared over the aerobic oxidation of HMF to FDCA in base free media. Among those catalysts, Ni_0.9_-Pd_0.1_/Mg(OH)_2_ resulted highest FDCA yield of 89% at 100°C. Improved FDCA production was obtained due to the activation of the hydroxyl group of HMF due to the basicity of support.

**Table 2:** HMF oxidation processes to FDCA by using O_2_ as the oxidant.

**Entry**	**Catalyst**	**Solvent**	**Oxygen pressure**	**Temp. (°C)**	**Reaction time (h)**	**FDCA yield (%)**	**HMF conversion (%)**	**References**
1)	Pt/C	NaOH	6.9 bar O_2_	22	6	79	100	Davis et al., 2011
2)	Pd/C	NaOH	6.9 bar O_2_	23	6	71	100	Davis et al., 2011
3)	Au/C	NaOH	6.9 bar O_2_	23	6	7	100	Davis et al., 2011
4)	Au/AC	NaOH	3 bar O_2_	60	6	80	>99	Villa et al., 2013
5)	Pd/AC	NaOH	3 bar O_2_	60	6	9	>99	Villa et al., 2013
6)	Pt/AC	NaOH	3 bar O_2_	60	6	20	>99	Villa et al., 2013
7)	Au/CNFs	NaOH	3 bar O_2_	60	6	6	>99	Villa et al., 2013
8)	Au/CNTs	NaOH	3 bar O_2_	60	6	39	>99	Villa et al., 2013
9)	Au/Graph	NaOH	3 bar O_2_	60	6	26	>99	Villa et al., 2013
10)	Au/C	NaOH	10 bar air	130	8	44	100	Casanova et al., 2009a
11)	Pt/RGO	NaOH	1 bar O_2_	25	24	84	100	Niu et al., 2014
12)	Fe_3_O_4_@C@Pt	Na_2_CO_3_	1 bar O_2_	90	4	100	100	Zhang et al., 2016
13)	Ru/C	NaHCO_3_	40 bar Air	100	2	75	100	Kerdi et al., 2015
14)	Ru/AC_NaOCl_	NaHCO_3_	40 bar Air	100	4	55	100	Kerdi et al., 2015
15)	Rh/C	pH = 13	10 bar O_2_	50	4	6.5	82	Vuyyuru and Strasser, 2012
1)	Au/CeO_2_	Na_2_CO_3_	5 bar O_2_	140	15	91	>99	Kim et al., 2018
2)	Au/Ce_0.8_Bi_0.2_O_2−δ_	NaOH	1 bar O_2_	65	2	>99	100	Miao et al., 2015
3)	Pt/TiO_2_	Na_2_CO_3_	1 bar O_2_	75	12	2	96	Sahu and Dhepe, 2014
4)	Pt/CeO_2_	Na_2_CO_3_	1 bar O_2_	75	12	8	100	Sahu and Dhepe, 2014
5)	Pt/C	Na_2_CO_3_	1 bar O_2_	75	12	89	100	Sahu and Dhepe, 2014
6)	Pt/ZrO_2_	Na_2_CO_3_	1 bar O_2_	75	12	94	100	Sahu and Dhepe, 2014
7)	Pt/Al_2_O_3_	Na_2_CO_3_	1 bar O_2_	75	12	96	96	Sahu and Dhepe, 2014
8)	Au/HY	NaOH	0.3 bar O_2_	60	6	>99	>99	Cai et al., 2013
9)	Au/TiO_2_	NaOH	0.3 bar O_2_	60	6	85	>99	Cai et al., 2013
10)	Au/Mg(OH)_2_	NaOH	0.3 bar O_2_	60	6	76	>99	Cai et al., 2013
1)	Au/TiO_2_	NaOH	10 bar O_2_	60	4	10	100	Pasini et al., 2011
2)	Cu/TiO_2_	NaOH	10 bar O_2_	60	4	-	100	Pasini et al., 2011
3)	Au-Cu/TiO_2_	NaOH	10 bar O_2_	60	4	30	100	Pasini et al., 2011
4)	Pd/TiO_2_	NaOH	10 bar O_2_	70	4	9	100	Lolli et al., 2015
5)	Au/TiO_2_	NaOH	10 bar O_2_	70	4	13	100	Lolli et al., 2015
6)	Pd-Au/TiO_2_	NaOH	10 bar O_2_	70	4	85	100	Lolli et al., 2015
7)	Pd/HT	NaOH	1 bar O_2_	60	6	6	85	Xia et al., 2019
8)	Au/HT	NaOH	1 bar O_2_	60	6	-	65	Xia et al., 2019
9)	Pd-Au/HT	NaOH	1 bar O_2_	60	6	90	100	Xia et al., 2019
10)	Au_8_-Pd_2_/C	NaOH	30 bar O_2_	60	4	>99	>99	Villa et al., 2013
11)	Pt/C	NaOH	1 bar O_2_	25	2	81	100	Verdeguer et al., 1993
12)	Pt-Pb/C	NaOH	1 bar O_2_	25	2	99	100	Verdeguer et al., 1993
13)	Pt/C	Na_2_CO_3_	40 bar air	100	6	69	99	Rass et al., 2013
14)	Pt-Bi/C	Na_2_CO_3_	40 bar air	100	6	>99	100	Rass et al., 2013
15)	Pd-Ni/Mg(OH)_2_	Base free	Air	100	16	89	>99	Gupta et al., 2017b
16)	Ru/C	HT	2 bar O_2_	120	5	60	100	Yi et al., 2016
17)	Ru/C	NaOH	2 bar O_2_	120	5	69	100	Yi et al., 2016
18)	Ru/C	K_2_CO_3_	2 bar O_2_	120	5	80	100	Yi et al., 2016
19)	Ru/C	Na_2_CO_3_	2 bar O_2_	120	5	93	100	Yi et al., 2016
20)	Ru/C	CaCO_3_	2 bar O_2_	120	5	95	100	Yi et al., 2016
21)	Au/HT-AC	Base free	5 Bar O_2_	100	12	>99	100	Gao et al., 2017
22)	Pt/ZrO_2_	Base free	4 bar O_2_	100	12	97.3	100	Chen H. et al., 2018
23)	Pt-Ni/AC	Base free	4 bar O_2_	100	15	97.5	100	Shen et al., 2018
24)	Pt-PVP-NaBH_4_	Base free	1 bar O_2_	80	24	80	100	Siankevich et al., 2014
25)	Pt-PVP-GLY^d^	Base free	1 bar O_2_	80	24	94	100	Siankevich et al., 2014
26)	Ru(OH)_x_/HT	Base free	1 bar air	140	24	19	99	Ståhlberg et al., 2012
27)	Ru(OH)_x_/La_2_O_3_	Base free	30 bar O_2_	100	5	48	98	Ståhlberg et al., 2012

**Scheme 5:**
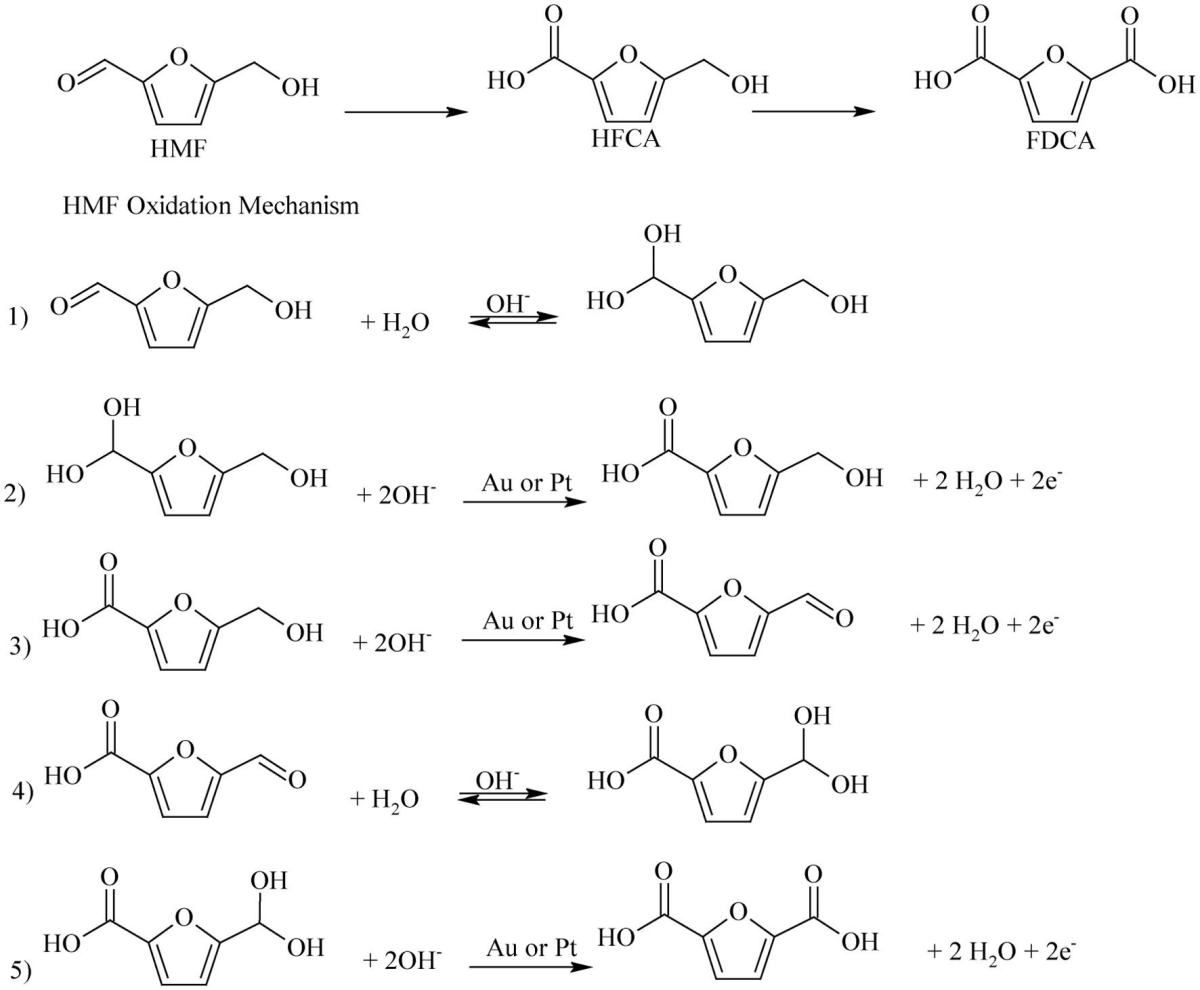
Reaction mechanism of HMF conversion to FDCA in alkaline media over Pt/Au catalyst. Reproduced with permission from Davis et al. (2012, 2014) copyright (2012) The Royal Society of Chemistry and (2014) Elsevier.

Most of the noble metal catalyzed aerobic oxidation of HMF to FDCA have been conducted under basic reaction media. Because, the basic environment allows the oxidization of the aldehyde group of HMF and keeps the formed FDCA dissolved in the solution. This prevents the adsorption of products on to the catalyst, allowing the activity of the catalyst to be maintained. The effect of the basicity of the reaction media over the oxidation of HMF to FDCA with Ru/C was investigated by Yi et al. (2016). Strong bases such as NaOH gave low FDCA yield (69%) in Ru/C catalyst, because of the degradation of HMF at high pH (Yi et al., 2016). K_2_CO_3_, Na_2_CO_3_, HT, and CaCO_3_ are weak bases and it was found that the latter gave the maximum yield of FDCA of 95%. The calcium salt of FDCA was formed due to its low stability when CaCO_3_ was used (Yi et al., 2016). In the basic environment the aerobic oxidation process proceeds either by alcohol group oxidation (Scheme 3, route A) or by aldehyde group oxidation (Scheme 3, route B). Davis et al. (2012, 2014) have investigated the reaction pathway for the aerobic oxidation of HMF to FDCA over Au/TiO_2_ catalyst with ^18^O_2_ isotope. Surprisingly no ^18^O_2_ isotope was found in the HFCA suggesting the hypothesis that the oxygen from water was utilized in the HMF oxidation reaction instead of using the oxygen gas which is the oxidant. This was confirmed by using labeled ${\text{H}}_{2}^{18}$O as the solvent and observed two isotopes of oxygen (^18^O_2_) in Na-HFCA and HFCA structures. As shown in Scheme 5, step 1, the aldehyde group of HMF side-chain is converted to the germinal diol through nucleophilic addition of hydroxide ion to the carbonyl group followed by proton transfer from water to the alkoxy ion intermediate. In the second step, the germinal diol dehydration occurred, and carboxylic acid and HMFCA formed from adsorbed hydroxide ions on the metal surface. Then, the alcoholic group of HMFCA formed an alkoxy intermediate by deprotonation of the alcoholic group (Zope et al., 2010). Regardless of the catalyst, base need to be present for the oxidation of alcoholic groups present in HFCA. Then, FFCA the aldehyde intermediate was formed as a result of the activation of the C-H bond in the alcoholic group by hydroxide ions on the catalyst's surface (Scheme 5, step 3). After that, in Scheme 5, steps 4 and 5, the aldehyde group of the FFCA is converted to FDCA. However, steps 4 and 5 are quite similar to steps 1 and 2 for the oxidation of HMF to HFCA. When the oxidation is done under ${\text{H}}_{2}^{18}$O, two more ^18^O atoms combine with FDCA due to the reversible hydration of the aldehyde group in step 4 to the geminal diol. At the end of the reaction, complete HMF oxidation to FDCA could be observed by the incorporation of four oxygen atoms in FDCA instead of O_2_. From the isotope labeling, the authors inferred that the oxygen atoms came from water and not from the oxidants. However, oxidizing agents indirectly facilitate the oxidative production of FDCA from HMF, by releasing the electrons deposited on the catalyst surface (Davis et al., 2012, 2014).

Even though, most of the noble metal incorporated catalysts are performed under aerobic oxidation of HMF to FDCA in basic media resulting in high yields of FDCA, when the environmental aspect is included in the equation, base-free oxidation systems are more desirable. Gupta et al. studied the reaction of Au/HT catalyst over the oxidation of HMF to FDCA at 95°C in water under 1 bar O_2_ pressure, and obtained a yield of 99% FDCA after 7 h. There was improved catalytic stability of HT with AC, so the catalyst, Au/HT-AC, which involved physical milling during preparation, resulted in 99.5% yield of FDCA (Gao et al., 2017). The slight improvement in yield is due to increased surface area, and higher proportions of basic sites and the existence of hydroxyl and carbonyl groups. Chen H. et al. (2018) have discussed the aerobic oxidation of HMF to FDCA by using Pt/ZrO_2_ catalyst in base-free conditions. After 12 h at 100°C temperature, 97.3% of FDCA yield was obtained with the complete conversion of HMF. The high yield of FDCA was due to the strong interaction between reactants and intermediates and excellent dispersion of Pt/ZrO_2_ catalyst with uniform particle size. Han et al. (2016a) have used a Pt catalyst with C-O-Mg support in base-free aerobic oxidation and observed 97% of FDCA yield at 110°C under 10 bar O_2_ pressure. Moreover, this catalyst could be used 10 times without significant loss of activity. Han et al. (2016b) have synthesized Pt/C-EDA-x catalyst (EDA = ethylenediamine and x = nitrogen dose) which is an N-doped carbon-supported Pt catalyst and used it at 110°C temperature for 12 h under 1 bar O_2_ pressure, and received 96% of FDCA yield. Apart from the monometallic catalysts, there is a lack of references regarding the bimetallic catalysts in base-free aerobic oxidation of HMF to FDCA. However, Shen et al. (2018) have reported that the catalytic activity of AC supported Pt-Ni bimetallic catalyst gave 97.5% FDCA yield with complete conversion of HMF at 100°C after 15 h under 4 bar O_2_ pressure with only 0.4% bimetal loading. The Pt-Ni bimetal catalyst gave good reusability and authors inferred that the addition of Ni to Pt increased the activity of Pt catalyst by improving the C=O adsorption and oxidation ability of Pt. Siankevich et al. (2014) have examined the base-free media with polyvinylpyrrolidone (PVP)-stabilized Pt catalyst for the HMF oxidation reaction under 1 bar O_2_ pressure for 24 h with 5 mol% of catalyst loading in water and observed 95% of FDCA yield. However, a slight decrease of FDCA yield was observed using Pt nanoparticle monometal catalyst (Pt-NPs). Pt-PVP catalyst has gained much attention as an oxidation catalyst in water because of its high oxidizing ability. Despite the potential of the Pt/PVP catalyst in terms of green environment protocol, it is not feasible economically because of its poor stability and recyclability.

**Scheme 6:**
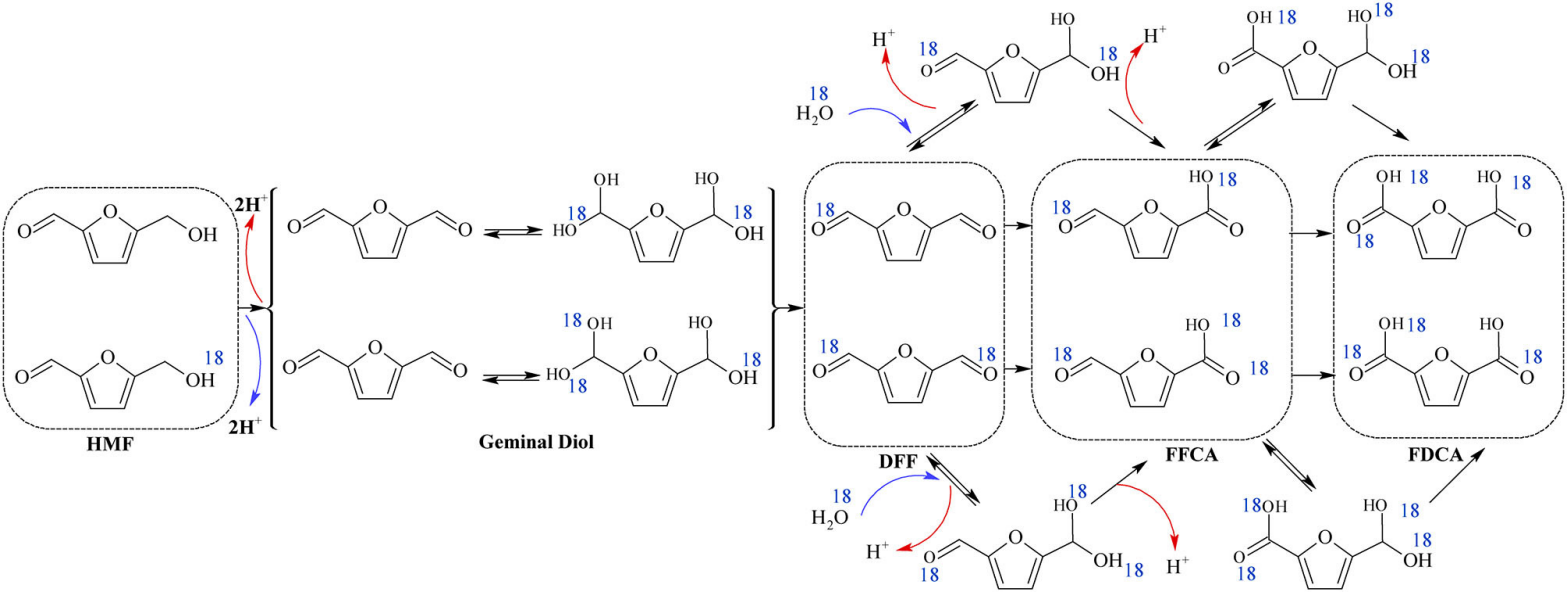
Reaction mechanism of HMF conversion to FDCA in base-free media with addition of ^18^O (blue). Resulted units are shown in dashed boxes. Reproduced with permission from Siankevich et al. (2014) copyright (2014) Elsevier.

Siankevich et al. (2014) have used isotope labeling method (${\text{H}}_{2}^{18}$O) to examine the base-free aerobic oxidation of HMF to FDCA. From isotope labeling study, it was established that base-free oxidation resulted in the formation of DFF as intermediate, and this mechanistic pathway have also been proposed by others (Davis et al., 2014; Wan et al., 2014). FFCA and FDCA were obtained as the products of HMF oxidation by Pt/PVP catalyst and the presence of ^18^O atoms were confirmed by the mass spectra (Siankevich et al., 2014). Similar to the step 1 of base oxidation mechanism as shown in Scheme 3, aldehyde groups were reversibly hydrated to germinal diol due to the nucleophilic addition of H_2_O to carbonyl groups as shown in the Scheme 6. As the final step, oxygen and hydrides are reacted together and migrate two protons to the metal surface and water and those two protons help for the regeneration of carboxylic groups (Siankevich et al., 2014).

Generally, organic solvents or water are used as the reaction media for Ru catalyst. However, Ståhlberg et al. (2012) have evaluated ionic liquids (ILs) in base-free media over Ru(OH)_x_ catalyst. ILs were selected as the reaction media because of their stability, low vapor pressure, non-flammability, and very high dissolving abilities. Among the various supports, La_2_O_3_ gave a reasonable FDCA yield of 48% with 98% HMF conversion at 100°C and 30 bar O_2_ pressure. However, HT supported Ru(OH)_x_ catalyst only 19% FDCA yield with 99% of HMF conversion at 140°C under 1 bar air pressure after 24 h. The reaction was carried out at a high temperature of 140°C to minimize the influence of viscosity of the IL (Ståhlberg et al., 2012). However, the work with ILs has lost interest because of the low FDCA yield, high price, and difficulty to scale-up.

In summary, basic reaction media have been successfully used in the aerobic oxidation of HMF to FDCA because they support the oxidation of aldehyde part of the HMF and allows FDCA to remain stable in the medium. However, base-free reaction media are more attractive because of environmental benefits as there is no need to neutralize the medium, and a second step to separate the resulting inorganic salts as a consequence of neutralization is not needed. Further work in this area is desired in order to fabricate better catalysts that can be used to process high HMF loading in order to increase the amount of FDCA produced.

#### Effect of Non-noble Metal Supported Heterogeneous Catalysts Over the Aerobic Oxidation of HMF to FDCA

Noble metal nanoparticles typically exhibit remarkable catalytic performance but due to their high cost, synthesis of non-noble metal catalysts have gained much attention. Promising results have been obtained with non-noble metals for the aerobic oxidation of HMF to FDCA as shown in Table 3 (Gao et al., 2015). Saha et al. (2013) synthesized a thermally stable catalyst by adding Fe^3+^ on the center of porphyrin ring supported on porous organic polymer (POP) and achieved 85% of FDCA yield at 100°C under 10 bar O_2_ pressure after 10 h in an aqueous reaction medium. The Fe-POP metal active site functions in a radical chain mechanism by thermal autoxidation of the organic material to peroxides. Wang S. et al. (2015) developed a non-noble metal catalyst (nano-Fe_3_O_4_-CoO_x_), which has magnetic properties and observed 69% FDCA yield with 97% HMF conversion at 80°C after 12 h. A mixture of mixed oxides of Fe_2_O_3_ and ZrO_2_ has been used for the base free oxidation of HMF to FDCA using ionic liquids (ILs) as solvents and co-catalysts (Yan et al., 2017, 2018b). This green and cost-effective route afforded about 60% FDCA yield at a near complete conversion of HMF, attributing the good performance of the Fe–Zr–O catalyst to the presence of large amount of acidic and basic sites on the catalyst surface as well as its high reducibility and oxygen mobility (Yan et al., 2018b), in combination with strong hydrogen bonding between the IL [Bmim]Cl and HMF which was favorable for the HMF oxidation to FDCA (Yan et al., 2017). Manganese (Mn) is another transition metal that has been used for the aerobic oxidation of HMF to FDCA. Hayashi et al. (2017) have investigated the HMF oxidation via MnO_2_ catalyst in NaHCO_3_ at 100°C under 10 bar O_2_ pressure and obtained 91% FDCA yield after 24 h. However, after the third cycle the catalyst was deactivated due to the covering of active sites by humin and the catalyst could be reactivated by calcining it at 300°C in air. The effect of the surface area in the FFCA oxidation to FDCA was discussed and it was observed that the highest catalytic activity was achieved by the β-MnO_2_ phase. Also, binary or mixed oxides have been used in the aerobic oxidation of HMF to FDCA, whereby Han et al. (2017) synthesized the MnO_x_-CeO_2_ (Mn/Ce = 6) catalyst and achieved 91% FDCA yield at 110°C temperature in KHCO_3_ media after 15 h. Structural analysis revealed that Mn^4+^ and Ce^3+^ ions functioned as active sites for HMF oxidation to FDCA. Zhang et al. (2018b) investigated the catalytic activity of the MnCo_2_O_4_ with a Mn/Co molar ratio of 1:2 over the conversion of HMF to FDCA at 100°C under 10 bar O_2_ pressure, and observed 70.9% FDCA yield with 99.5% HMF conversion after 24 h. Improved catalytic activity was caused due to the Mn^3+^ and increased oxygen mobility and reducibility.

**Table 3:** Aerobic HMF oxidation to FDCA over non-noble and metal-free catalysts.

**Entry**	**Catalyst**	**Solvent**	**Oxygen pressure**	**Temp. (°C)**	**Reaction time (h)**	**FDCA yield (%)**	**HMF conversion (%)**	**References**
1)	Fe-POP	-	10 Bar O_2_	100	10	79	100	Saha et al., 2013
2)	Fe_3_O_4_-CoO_x_	-	*t*-BuOOH	80	12	68.6	97.2	Wang S. et al., 2015
3)	Ce_0.5_Fe_0.5_O_2_	[Bmim]Cl	20 bar O_2_	140	24	13.8	98.4	Yan et al., 2017
4)	Ce_0.5_Zr_0.5_O_2_	[Bmim]Cl	20 bar O_2_	140	24	23.2	96.1	Yan et al., 2017
5)	Ce_0.5_Fe_0.15_Zr_0.35_O_2_	[Bmim]Cl	20 bar O_2_	140	24	44.2	99.9	Yan et al., 2017
6)	Fe_0.6_Zr_0.4_O_2_	[Bmim]Cl	20 bar O_2_	160	24	60.6	99.7	Yan et al., 2018b
7)	MnO_2_	NaHCO_3_	10 bar O_2_	100	24	91.0	>99	Hayashi et al., 2017
8)	MOF-Mn_2_O_3_	NaHCO_3_	14 bar O_2_	100	24	99.5	100	Bao et al., 2019
9)	MnO_x_-CeO_2_	KHCO_3_	20 bar O_2_	110	15	91	98	Han et al., 2017
10)	MnCo_2_O_4_	KHCO_3_	20 bar O_2_	100	24	70.9	99.5	Zhang et al., 2018b
11)	Co_3_O_4_/Mn_x_Co	Base free	14 bar O_2_	140	24	>99	100	Gao et al., 2019
12)	NNC-900	3 equiv. K_2_CO_3_	1.0 bar O_2_	80	48	80	100.0	Nguyen et al., 2016
13)	NNC-873	3 equiv. K_2_CO_3_	1.0 bar O_2_	80	48	11	100.0	Nguyen et al., 2016
14)	NNC-973	3 equiv. K_2_CO_3_	1.0 bar O_2_	80	48	35	100.0	Nguyen et al., 2016
15)	NNC-1073	3 equiv. K_2_CO_3_	1.0 bar O_2_	80	48	72	100.0	Nguyen et al., 2016
16)	NNC-1173	3 equiv. K_2_CO_3_	1.0 bar O_2_	80	48	80	100.0	Nguyen et al., 2016

So far, base-free aerobic oxidation of non-noble metal catalysts has not received much attention. However, recently Gao et al. (2019) reported the synthesis of Co_3_O_4_/Mn_0.2_Co with a Mn:Co ratio of 0.2 and obtained >99% of FDCA yield at 140°C under 1 bar O_2_ for 24 h. Excellent adsorption of HMF and COOH formation occurred on the Lewis acid (Mn^4+^) and Brφnsted (Co-O-H) acid sites of the catalyst.

In summary, recent work has shown that non-noble metal catalysts can have similar catalytic activity as noble metal catalysts in the aerial oxidation of HMF to FDCA. However, metal leaching, blockage of the active sites and changing the active phases during reactions are some of the adverse effects that affect the catalytic performance of non-noble metal catalysts in the oxidation of HMF to FDCA. From cost benefit point of view, further investigations should be conducted to design efficient non-noble metal catalyst that can provide economic routes to selectively produce FDCA.

#### Effect of Metal-Free Heterogeneous Catalysts Over the Aerobic Oxidation of HMF to FDCA

The high cost of using noble metal catalysts, and to a lesser extent transition metal catalyst, as well as metal ion leakage into the reaction medium (resulting in purification issues of the product) has hindered the development of industrial applications for FDCA production from HMF. Nguyen et al. (2016) has developed a metal-free, high nitrogen-doped nanoporous graphitic carbon (NNC) catalyst for effective conversion of HMF to FDCA. Unlike conventional nitrogen-doped carbon materials, a zeolite imidiazole framework was designed for the NNC materials. Various carbonization temperatures were evaluated and the carbonization carried out at 900°C (and 1,173°C) has resulted in the highest FDCA yield of 80% with 100% conversion of HMF at 80°C in alkaline media by using oxygen as the oxidant. Also as shown in Table 3, NNC-873, NNC-973, and NNC-1073 gave 11, 35, and 72% of FDCA yield, respectively, implying that the temperature of carbonization influenced HMF oxidation to FDCA (Nguyen et al., 2016). There was a proportional relationship between FDCA yield and graphene-like nitrogen structure, and it was suggested that the nitrogen atoms on the structures were the main active sites for the activation of oxygen that gives the components of oxygen radicals. However, for the NNC-1173 catalyst, a reduced amount of graphene type nitrogen resulted in higher yield of FDCA (Nguyen et al., 2016). The reason for this is not known. These NNC catalysts have also shown ease of separation by centrifugation and the ability to recycle in multiple runs (only 10% decrease of FDCA in the fourth run without any structural changes) by showing their suitability as heterogeneous catalysts in the aerobic oxidation of HMF to FDCA (Nguyen et al., 2016). However, increased reaction time and low ratio between HMF and base additive limit the use of NNC catalysts in industrial applications. Therefore, further investigations need to be conducted to make this green concept of metal-free aerobic oxidation of HMF to FDCA competitive in industrial applications.

### H_2_O_2_ as an Oxidant

Hydrogen peroxide is a liquid oxygen source, an alternative that has been used in industries in large scale due to its reasonable price, high active-oxygen content, and ability to store safely (Rinsant et al., 2014). It is used as an oxidizing agent in different reactions such as Baeyer–Villiger oxidation reaction, (Ten Brink et al., 2004) alcohol oxidation, (Han et al., 2015), and phenol hydroxylation (He et al., 2015). Also, hydrogen peroxide has been used as an oxidant in the oxidation of glucose to gluconic acid (Rinsant et al., 2014), levulinic acid to succinic acid (Dutta et al., 2015a) and furfural to maleic or fumaric acid (Araji et al., 2017). In most studies, O_2_ is used as an oxidant in the oxidation reaction of HMF to FDCA. However, the need of high O_2_ pressure in the oxidation of HMF to FDCA raises safety issues. Therefore, H_2_O_2_ which produces O_2_ and water was tested in place of O_2_ in the oxidation of HMF to FDCA (Chen C. T. et al., 2018).

Chen C. T. et al. (2018) reported a high yield of 91% FDCA when a molar ratio of 10 between HMF and Ru/C was used in Na_2_CO_3_ solution at 75°C and a reaction time of 6 h with H_2_O_2_ as the oxidant. FFCA was found to be the intermediate product in this reaction. With HMF/Ru molar ratio of 50 in NaHCO_3_ solution, 91.3% FDCA yield was obtained, perhaps indicating the benefits of working in a stronger basic medium. The reaction system used by Chen et al. allowed HMF to be oxidized in water under mild reaction conditions efficiently with H_2_O_2_, as it was shown that the use of H_2_O_2_ is a lower mass transfer resistance than gaseous oxygen-assisted oxidation systems.

Microwave assisted HMF oxidation to FDCA has been studied in batch and continuous reactors by Zhao et al. (2020b). They obtained high FDCA yield of 88% after 30 min with the commercial catalyst 5% Ru/C in a batch reactor system (Zhao et al., 2020b). Zhao et al. (2020a) have further investigated HMF oxidation in batch reactor system via microwave heating with silver oxide heterogeneous catalyst and obtained 97% HMFCA after 24 h. In another study, Zhao et al. (2020b) in a microwave assisted continuous flow oxidation process obtained 47% of FDCA yield in the presence of H_2_O_2_ as an oxidant. Ji et al. (2018) conducted HMF oxidation under microwave irradiation with 3% Au/TiO_2_ in the presence of H_2_O_2_. As working in an alkaline medium accelerates the conversion process, by increasing the pH from 9 to 14, wherein H_2_O_2_ and NaOH are added in a continuous mode to the reactor, the conversion of HMF to the intermediate, HMFCA was increased 100-fold, and FFCA to FDCA by 66-fold (Ardemani et al., 2015). An overall FDCA yield of >99% after 30 min, at atmospheric pressure and <100°C was obtained (Ji et al., 2018). Apart from the effect of catalysts Zhang and Zhang (2017) investigated the synthesis of FDCA from HMF oxidation by using alkaline media HMF:KOH:H_2_O_2_ in the ratios of 1:4:8 and obtained 55.6% of FDCA yield after 15 min of reaction at 70°C temperature.

As a substitute for the noble metal catalyzed HMF oxidation reaction, Martínez-Vargas et al. (2017) immobilized salen complexes of Co(II), Fe(II) and Cu(II) onto SBA-15 (Table 4) to study reaction mechanism for the oxidation of HMF by H_2_O_2_ at room temperature (25°C) to FDCA. The study found that the oxidation route of alcohol group in HMF is favored over the oxidation of the aldehyde group on the other side of the molecule.

**Table 4:** HMF oxidation processes to FDCA by using H_2_O_2_ as the oxidant.

**Entry**	**Catalyst**	**Solvent**	**Temp. (°C)**	**Reaction time (h)**	**FDCA yield (%)**	**HMF conversion (%)**	**References**
1)	Molybdenum complex [EMIM]_4_Mo_8_O_26_	Aqueous NaOH	100	2.00	99.5	-	Li et al., 2016
2)	Au/TiO_2_	Aqueous NaOH	97	0.50	>99.0	-	Ji et al., 2018
	-	KOH	70	0.25	55.6	-	Zhang and Zhang, 2017
3	Co(II), Fe(III), Cu(II) salen/SBA-15	-	25	1.66	<10.0	99%	Martínez-Vargas et al., 2017
4)	Ru/AC	NaHCO_3_	75	6.00	92.0	-	Chen C. T. et al., 2018
5)	Ru/AC	Na_2_CO_3_	75	1.00	91.3	-	Chen C. T. et al., 2018

### Other Oxidants

Researches have been conducted by using various oxidants instead of O_2_ and H_2_O_2_ in aspect of the oxidation reaction of HMF to FDCA. Because, H_2_O_2_ is a highly active oxidant and needs to be handled carefully, it is less favorable from an industrial aspect. The catalytic activity of O_2_ is relatively low compared to other oxidants (H_2_O_2_, *t*-BuOOH) and O_2_ needs high pressure to facilitate the oxidation of HMF to FDCA which could cause issues in energy consumption and production costs. Gao et al. (2015) reported the use of Merrifield resin-Co-Py catalyst with different oxidants in the HMF oxidation reaction to FDCA as shown in Table 5. The oxidant sodium periodate (NaIO_4_) showed the highest HMF conversion (96.5%) but the yield of FDCA was very low (3.9%). The reason for this could be the high oxidative property of NaIO_4_ causing the destruction of HMF furan rings (Gao et al., 2015). When, *t*-BuOOH was used as the oxidant 60.3% yield of FDCA was obtained with 77.5% conversion of HMF after 24 h of reaction at 70°C whereas, at 40°C temperature FDCA yield was 6.6%. This suggested the temperature dependence of Merrifield resin-Co-Py catalyst in *t*-BuOOH oxidant (Gao et al., 2015). Also, the Merrifield resin-Co-Py catalyst in CH_3_CN solvent under *t*-BuOOH oxidant yielded 88.1% FDCA after 48 h with 97.2% HMF conversion (Gao et al., 2015). As an oxidant, *t*-BuOOH has advantages for usage in catalytic reactions due to its availability, the ability for usage in large scale synthesis, and moderate reactivity on organic substances (Gao et al., 2015).

**Table 5:** HMF oxidation processes to FDCA by using other oxidants.

**Entry**	**Catalyst**	**Oxidant**	**Temp. (°C)**	**FDCA yield (%)**	**HMF conversion (%)**	**References**
1)	CuCl_2_	*t*-BuOOH	25	45.0	100.0	Hansen et al., 2013
2)	Fe_3_O_4_-CoO*_*x*_*	*t*-BuOOH	80	68.6	97.2	Zhang et al., 2015
3)	Merrifield resin-Co-Py	*t*-BuOOH	100	90.4	95.6	Gao et al., 2015
4)	Merrifield resin-Co-Py	NaIO_4_	70	3.9	96.5	Gao et al., 2015
5)	Merrifield resin-Co-Py	*t*-BuOOH	40	6.6	17.2	Gao et al., 2015
6)	CH_3_CN	*t*-BuOOH	70	88.1	97.2	Gao et al., 2015

### Enzymatic Oxidation of HMF to FDCA

The conversion of HMF to FDCA via enzymes is more advantageous than chemical conversion due to the ability of enzymes to be active under mild conditions (Sajid et al., 2018). When in search of green catalytic conversions, biocatalytic conversions are found the most suitable due to their non-hazardous byproducts and intermediates. However, the biocatalytic pathway to FDCA production from HMF has not been well-identified. Generally, enzymes are either specific to the aldehyde group or to the alcohol group, whereas HMF oxidation to FDCA requires the oxidation to take place both sites. Chloroperoxidase is one biocatalyst that was used for HMF oxidation with H_2_O_2_ and was able to produce mainly DFF (60–74%), and smaller amounts of HFCA and FFCA (Van Deurzen et al., 1997). A chloroperoxidase obtained from *Caldariomyces fumago* has the ability to oxidize HMF to FDCA. However, the FDCA yield is relatively low (60–75%) and due to the difficulty to obtain chloroperoxidase in the pure form from *C. fumago* is not suitable for the bioconversion of HMF to FDCA (Sajid et al., 2018).

**Scheme 7:**
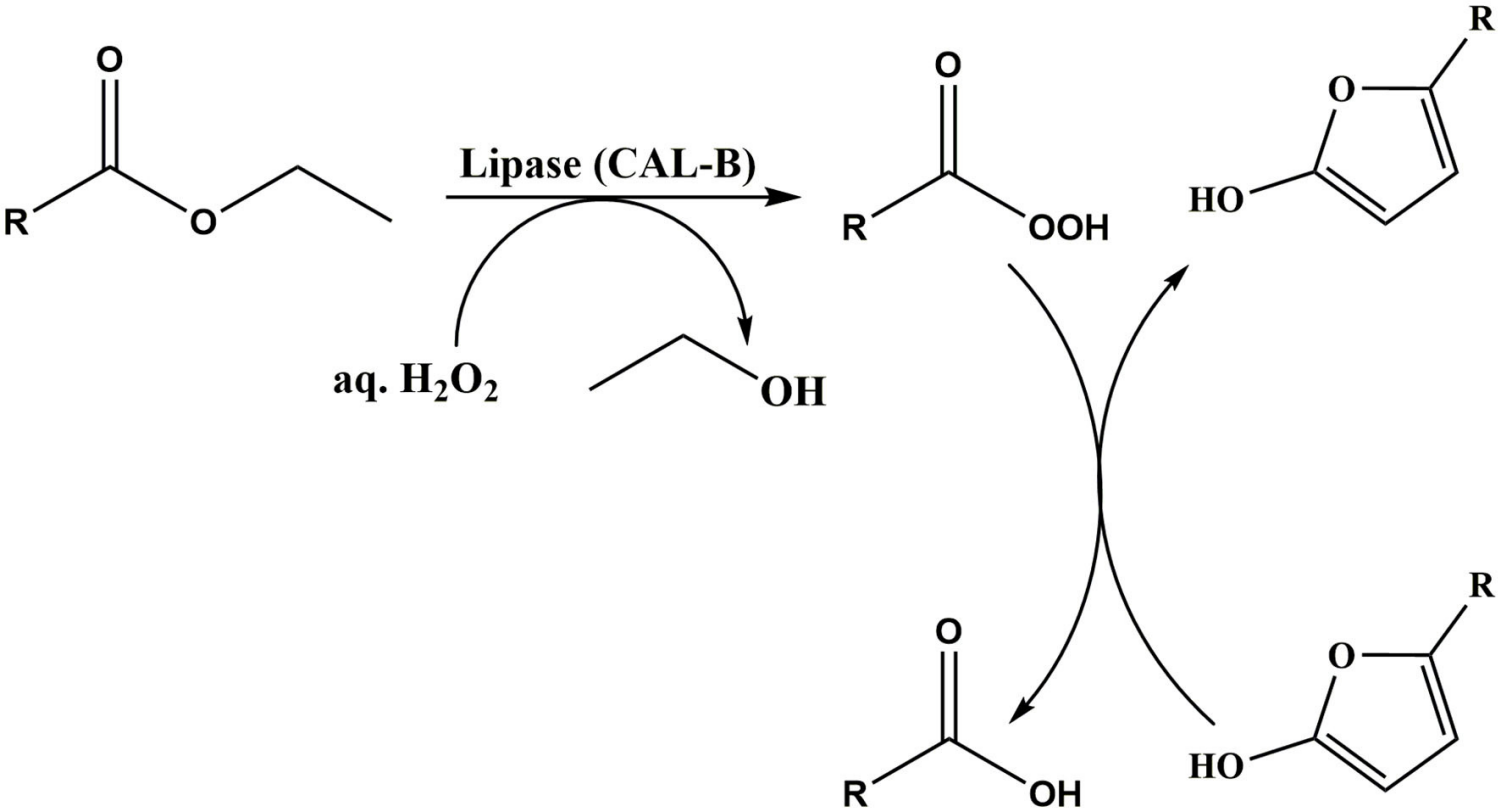
HMF conversion to FDCA by *in situ* prepared organic peracid. Reproduced with permission from Krystof et al. (2013) copyright (2013) John Wiley and Sons.

The unique enzyme, hexamethyl furfural oxidase (HMFO) which was identified to attack both the aldehyde, and alcohol groups was shown to convert HMF to 95% FDCA yield (Dijkman et al., 2015). Karich et al. (2018) has studied the enzymatic oxidation of HMF using wild type aryl alcohol oxidase (AAO), wild type peroxygenase (UPO) and recombinant galactose oxidase (GAO), and achieved a 80% FDCA yield. More recently, HMF to FDCA oxidation has been studied in one-pot two-step approach by Krystof et al. (2013). As shown in Scheme 7, initially, silica immobilized 2,2,6,6-tetramethylpiperidine-1-oxyl (TEMPO) used to selectively oxidize HMF to DFF and obtained 23% yield. Then, DFF was further oxidized to FDCA at 40°C for 24 h by using peracetic acid which was *in situ* prepared with lipase by adding 30% H_2_O_2_ and >99% of FDCA yield has been obtained (Krystof et al., 2013). However, conversion of HMF to FDCA in one-pot two step is still in its infancy stage and further investigations need to be done to make this biocatalytic oxidation more economical (Krystof et al., 2013). However, the drawbacks with enzymatic oxidation include lengthy reaction-time (≥24 h) and the low feedstock concentration requirement (Zhang and Deng, 2015).

### Electrochemical Oxidsation of HMF to FDCA

Electrochemical oxidation of HMF offers an alternative route for FDCA production with the advantage that the oxidant is replaced by electrons thereby reducing overall waste from the process. Unfortunately low yields are achieved and product recovery remains an issue especially when employed in a base medium (Sajid et al., 2018). Grabowski et al. (1991) initially investigated H-shaped electrochemical cells for the oxidation of HMF to FDCA by using nickel oxide/hydroxide as the anode and stainless steel as the cathode with NaOH as the alkaline medium. The yield of FDCA was 71% after 4 h at the anode. Chadderdon et al. (2014) have evaluated Au/Carbon black in alkaline media over the electrochemical conversion of HMF. Only aldehyde oxidation occurred as higher electrochemical potential is required for alcohol oxidation (Chadderdon et al., 2014). Recently, electrochemical oxidation in acidic media has emerged as a means to overcome product recovery as FDCA can precipitate out from the solution. In this process by Kubota and Choi (2018), FDCA yields of 53.8% were achieved but with a significant by-product formation. Vuyyuru and Strasser (2012) have used Pt electrodes and +0.44 mAcm^−2^ current density over the electrochemical HMF oxidation to FDCA. However, only 18% FDCA was recorded with 70% HMF conversion due to the competitive electrolysis of water to oxygen and hydrogen. While promising, the scale-up of electrochemical methods to larger scale production is also an engineering challenge.

## Conversion of Sugars Directly to FDCA

As the search of green and sustainable technologies for the production of FDCA, direct conversion of renewable lignocellulose biomass or biomass derived sugars to FDCA is important. Moreover, the use of environmental benign organic solvents and catalysts will make the FDCA synthesis from sugars more sustainable (Sheldon, 2014). Biochemical production of FDCA directly from sugars is a green and sustainable method compared to chemical catalytic conversion processes. However, as discussed in section Heterogeneous Catalytic Oxidation of HMF to FDCA, the use of microorganisms and enzymes have only been investigated for HMF oxidation to FDCA. With regards to the direct conversion of biomass to FDCA, simple sugars have been mainly explored because of ease of conversion. Among the sugars, fructose is more widely used in the production of FDCA due to its furanose structure which is structurally more appropriate for formation of furan products. To effectuate the conversion of sugars to FDCA using solid catalysts, both acid and metal sites must be present. Conversion of sugars to FDCA can be studied either in a single or multiphase media.

### One-Pot Two-Steps in Single Phase Reactions

One-pot methods involving two-steps have been vastly studied in the conversion of fructose to FDCA as shown in Table 6, and combine the benefits of high FDCA yield and catalyst recoverability. Using dual nanocatalysts, Fe_3_O_4_@SiO_2_-SO_3_H catalyst produced 93.1% HMF from fructose which was *in-situ* oxidized to FDCA in 59.8% yield using nano-Fe_3_O_4_-CoO_x_ catalyst and *t*-BuOOH is used as the oxidant (Wang S. et al., 2015). This method provides benefits due to the ease of separation and recyclability of Fe_3_O_4_-CoO_x_ catalyst by utilizing its magnetic property and an alkaline solvent was not used. Fructose conversion to FDCA in iPrOH/H_2_O or tetrahydrofuran (THF)/H_2_O results rapid production of HMF from fructose (Yi et al., 2016). Base-free conditions were used in the subsequent oxidation reaction by Ru/C catalyst and an overall 53% yield of FDCA after 15 h reaction time was obtained. Use of low-cost Ru non-precious metal catalyst and amenable reaction conditions to product and catalysts separation makes this method promising.

**Table 6:** Summary of the one-pot two-step methods for the production of FDCA from fructose.

**Synthesis of HMF**	**HMF yield/%**	**Synthesis of FDCA**	**FDCA yield/%**	**References**
Catalyst: Lewatit SPC 108 Temp.: 80°C Time: 30 min Initial concentration: 10 g/l Solvent: water	80	Catalyst: Silicon encapsulated—PtBi/C Oxidant: air oxygen Temp.: 80°C Time: 30 min Initial concentration: 10 g/l Solvent: MIBK	25	Kröger et al., 2000
Catalyst: Amberlyst-15 Temp.: 95°C Time: 30 min Initial concentration: Solvent: TEAB/H2O phase	86	Catalyst: Au8Pd2/HT Oxidant: oxygen Temp.: 95°C Time: 420 min Initial concentration: Solvent: Water	78	Yi et al., 2015
Catalyst: SiO2 gel Temp.: 88°C Time: 480 min Initial concentration: Solvent: MIBK	47	Catalyst: Co(acac)3 Co-gel Oxidant: air Temp.: 160°C Time: 65 min Initial concentration: Solvent: Water	46 [Co(acac)3] 72 (Co-gel)	Ribeiro and Schuchardt, 2003
Catalyst: Fe3O4@SiO2–SO3H Temp.: 100°C Time: 120 min Initial concentration: Solvent: DMSO	93	Catalyst: nano-Fe_3_O_4_-CoO_x_ Oxidant: *t*-BuOOH Temp.: 80°C Time: 720 min Initial concentration: Solvent: DMSO	68	Wang S. et al., 2015
Catalyst: HCl Temp.: 180°C Time: Initial concentration: Solvent: γ-valerolactone (GVL)/H2O	70	Catalyst: Pt/C Oxidant: oxygen Temp.: RT Time: 1,200 min Initial concentration: Solvent: GVL/H2O	93	Motagamwala et al., 2018
Catalyst: Pd/C Temp.: 140°C Time: 540 min Initial concentration: Solvent: water	85	Catalyst: Pd/CC Oxidant: oxygen Temp.: 140°C Time: 1,800 min Initial concentration: Solvent: water + K_2_CO_3_	64	Rathod and Jadhav, 2018
Catalyst: Fe3O4–RGO–SO3H Temp.: 110°C Time: 120 min Initial concentration: Solvent: DMSO	81	Catalyst: ZnFe_1.65_Ru_0.35_O_4_ Oxidant: Oxygen Temp.: 130°C Time: 960 min Initial concentration: Solvent: water + DMSO	70	Yang et al., 2016
Catalyst: Amberlyst-15 Temp.: 120°C Time: 60 min Initial concentration: 5 wt% Solvent: DMSO	97	Catalyst: Pt/C catalyst Oxidant: O_2_ Temp.: 100–120°C Time: 600 min Initial concentration: Solvent: K_2_CO_3_ /H_2_O/DMSO	88	Chen G. et al., 2018

Conversion of fructose to FDCA using Pd/CC catalyst was able to achieve 64% FDCA yield (Rathod and Jadhav, 2018). The benefit of Pd/CC catalyst is that due to its high surface area and multiple acid functional groups like: –SO_3_H, –OH, –COOH and –NH_2_; it can both dehydrate fructose to HMF and due to its oxidative sites, oxidize HMF to FDCA. Another green approach for the production of FDCA from fructose was studied using Pt/C catalyst and γ-valerolactone (GVL) as co-solvent with water (Motagamwala et al., 2018). In this approach, the product FDCA is used as the acid catalyst for the dehydration of fructose and Pt/C catalyst then oxidizes HMF to FDCA. Interestingly, GVL can be made from levulinic acid, which is a by-product of the HMF to FDCA reaction and represents a more sustainable example of reutilizing waste from the process to make the solvent. This method is industrially and environmentally feasible due to lower costs as there is limited requirement for external catalyst and solvent as they can be produced in the process or derived from the byproducts.

Bifunctional catalyst cobalt acetylacetonate (Co(acac)_3_) cooperated sol-gel silica was used to convert fructose to FDCA, with 99% selectivity and 72% conversion obtained due to the synergistic effect of acidic and metal sites (Ribeiro and Schuchardt, 2003). The dehydration of fructose occurred at the acidic sites of the silica surface and the oxidation occurred inside the silica pore channels by the action of the metals in it (Ribeiro and Schuchardt, 2003). This diffusion of HMF into the matrix could be increased at higher temperatures and pressure. This catalytic system shows high selectivity by forming no byproducts. This phenomenon could have occurred due to the rapid conversion of the intermediate HMF preventing the degradation to levulinic and formic acids. In the conversion of fructose to FDCA, using nano-Fe_3_O_4_-CoO_x_ catalyst produced DFF and HMFCA as the intermediates (Wang S. et al., 2015) confirming the oxidation of both aldehyde and hydroxyl group of HMF. Another magnetic catalyst used to convert fructose to FDCA is ZnFe_1.65_Ru_0.35_O_4_ (Yang et al., 2016). In the first step, Fe_3_O_4_-RGO-SO_3_H is used in an air atmosphere at 110°C in DMSO solvent and 81.3% of HMF yield was obtained after 2 h. Then, Fe_3_O_4_-RGO-SO_3_H was magnetically removed and oxidation step conducted in O_2_ atmosphere at 130°C by adding water and ZnFe_1.65_Ru_0.35_O_4_, which produced a yield of 70.5% FDCA after 16 h. In another study, sucrose was converted to FDCA, with 34% yield of HMF obtained after hydrolyzation and dehydration of sucrose using sulfuric acid in a tubular reactor at 200°C under 25 bar pressure (Schade et al., 2019). Au/ZrO_2_ catalyst used to oxidize the resulting solution obtained an overall FDCA yield of 29%.

The use of HCl as a catalyst in isopropanol resulted in 84% yield of HMF after the dehydration of fructose and Au/HT catalyst resulted in a subsequent 83% FDCA yield by oxidation of HMF (Yi et al., 2014). Benefits of this process include efficient isopropanol recovery and high FDCA yield. Also, this process revealed the most economical purification method with 98% yield by water extraction after the oxidation over Au/HT catalyst. Also, HCl and AlCl_3_ was used in the process of converting glucose to FDCA in water/THF biphasic system and obtained a yield of 52% FDCA. After successful production of FDCA from fructose and glucose, conversion of biomass to FDCA was then studied with Jerusalem artichoke (JAT), selected because of its high inulin/fructose content, abundancy, and fast growing nature (Yi et al., 2014). Conversion of JAT to FDCA was performed in water/ methylisobutyl ketone (MIBK) biphasic system allowing high retention of impurities, biomolecules, ions, fibers, and gels, in the water phase and HMF was efficiently extracted into the MIBK phase achieving a 57% yield. After MIBK evaporation, HMF was extracted into water and oxidized using Au/HT catalyst to achieve 55% overall FDCA yield (based on fructose content in JAT).

A direct one-pot, two-step conversion of inulin to FDCA was also studied where, inulin was first converted to HMF in KBr (30 mol%) and H_2_SO_4_ catalyst (Wrigstedt et al., 2017). The resultant HMF was then oxidized using Pt/C catalyst under NaHCO_3_ basic medium at 80°C and 14 h reaction time, with 44% yield of FDCA obtained. In another study, Amberlyst-15 and Fe_0.6_Zr_0.4_O_2_ were used as the oxidation catalysts in the conversion of fructose to FDCA in [BMIM][Cl] ionic liquid and observed 46.4% of FDCA yield (Yan et al., 2018a). IL only by itself has resulted 11.4% FDCA yield at 160°C after 24 h of HMF oxidation. Rapid conversion of fructose to HMF is achieved with Amberlyst-15 in high yield (>83%) in 1 min providing a solid basis for the subsequent high yield of FDCA. Initially fructose dehydrated to HMF and then HMF oxidized to 5-hydroxymethylfuranoic acid (HMFA) instead of DFF (Yan et al., 2018a). Then, HMFA oxidized to FFCA and FDCA confirming the reaction mechanism is similar to the general fructose conversion to FDCA mechanism. Rate constants were determined for each reaction step. The production of FDCA from fructose is considered as a first order reaction (Qi et al., 2009; Yang et al., 2016). Yan and the team considered the effect of different reaction parameters for the production of FDCA and calculated activation energy values for fructose to HMF conversion (59.7 kJ/mol), HMF to HMFA oxidation (82.7 kJ/mol), HMFA to FFCA oxidation (86.4 kJ/mol), and FFCA to FDCA oxidation (110.2 kJ/mol) (Yan et al., 2018a).

In the sucrose conversion to FDCA, 34% yield of HMF obtained after hydrolyzation and dehydration to HMF by using sulfuric acid in a tubular reactor at 200°C under 25 bar pressure (Schade et al., 2019). Au/ZrO_2_ catalyst used to oxidize the resulted solution in the first step and obtained overall FDCA yield of 29%.

### Multiphase Reactions

For multiphase reaction systems, dehydration of fructose to HMF occurs in one medium then oxidation of HMF to FDCA occurs in another medium. Acid-catalyzed formation of HMF followed by *in situ* oxidation as a single process can minimize the loss of HMF. If *in situ* oxidation occurs in the same phase, fructose can also be oxidized by the catalyst. Kröger et al. (2000) proposed water/MIBK system and was used polytetrafluoroethylene membrane to separate the water and MIBK phases. HMF yield of 12% was observed in aqueous phase by using sulfonic resin, Lewatit SPC 108 microporous catalyst and oxidation was occurred in the MIBK solution. When only MIBK as a single phase reaction was used, HMF oxidation produced only DFF. Adding water as a co-solvent to MIBK allowed the production of FDCA. When MIBK was saturated with water, the yield of all the products: 2,5-furandialdehyde (FDA), FFCA, and FDCA increased (Kröger et al., 2000). However, FDCA yield decreased when a high amount of levulinic acid (25%) was able to diffuse through the membrane. Even though the overall FDCA yield is quite low, this work opens a new pathway for further research. In considering the kinetics, initially fructose dehydrates to HMF by removing water molecules and then levulinic acid, humins, and formic acids are subsequently formed (Kröger et al., 2000). The oxidation steps are not rate limiting, rather the total reaction rate depends on the diffusion of HMF through the membrane and the competing formation of byproducts like levulinic acid in the aqueous phase. In a second approach, the *in situ* oxidation was achieved using PtBi/C catalyst encapsulated in silicone beads to selectively oxidize only HMF and not fructose and received 25% FDCA with 50% selectivity (Kröger et al., 2000).

Apart from biphasic systems, a triphasic system has also been used in the conversion of sugars to FDCA. Tetraethylammonium bromide (TEAB) was used as phase I and dehydration of sugars occurred in this phase using Amberlyst-15 catalyst (Yi et al., 2015). Phase III consists of water wherein HMF oxidation to FDCA took place on Au/HT catalyst while phase II (MIBK) serves to transport HMF formed in phase I to phase III. By using this triphasic reaction system, high yield of FDCA (78%) was obtained from fructose, although the yield reduced to 50% when glucose was used as the feedstock (Yi et al., 2015). The limiting step of this reaction was transferring HMF from phase I to III. Also, in phase III only HFCA and FDCA were present while high amount of HMF was present in phase II. This confirms the HMF oxidation to FDCA occurs via HFCA and conversion of HMF to HFCA occurs very rapidly. In the production of FDCA from glucose, in triphasic reactor Amberlyst-15/CrCl_3_ was used as catalysts. CrCl_3_ was selected as the catalyst due to its ability to isomerize glucose to fructose. Also, low reaction temperature led to the lower HMF yield in phase I and to an overall low FDCA yield (Yi et al., 2015).

## Production of FDCA Derivatives

The catalytic oxidation of HMF to FDCA is a process with high FDCA yield in solution, though there is not much work on its recovery and purification. When excessive base is used, FDCA converts into its salt and byproducts which makes it difficult to obtain purified FDCA. Therefore, synthesis of platform chemical 2,5-furandicarboxylic acid dimethyl ester (FDCDM) has gained attention as FDCDM can be easily converted to FDCA by simple hydrolysis reaction after purification via vacuum distillation. In comparison to FDCA, when making polyesters, the use of diethyl or dimethyl esters of FDCA exhibit lower melt temperature and are normally more miscible in the corresponding diols. This is particularly the case for enzyme catalyzed polymerisations, such as the examples of work by Jiang et al. (2015, 2016). Parisi (2019) has also shown the role that enzymes can play in making polymer precursors (bis-diols) from the dimethylester.

Taarning et al. (2008) have reported the one-pot esterification of HMF to FDCDM with 98% yield using Au/TiO_2_ catalyst at 130°C in MeOH with the presence of 8% MeONa, where, MeONa was used to ensure the reaction proceeds to completion. Conversion of HMF to FDCDM projects similarities with HMF oxidation to FDCA in water over Au catalysts (Casanova et al., 2009a). Where, fast oxidative esterification results in 5-hydroxymethyl methylfuroate (HMMF) formation, this product then more slowly converts to FDCDM. Casanova et al. (2009b) investigated the effect of base-free environment for the oxidative esterification of HMF to FDCDM using Au/CeO_2_ catalyst and achieved 99% yield at 130°C temperature and 10 bar O_2_ pressure. However, the need of 8% MeONa for the smooth catalytic reaction of Au/TiO_2_ suggests Au/CeO_2_ has higher catalytic activity than Au/TiO_2_ due to the production of peroxo and superoxo groups in nanocrystalline ceria. According to the reaction kinetics of Au/CeO_2_ catalyst, the rate limiting step was the oxidation of alcohol groups. Advantages of using Au/CeO_2_ catalyst are the ease of recovery and reusability, whereas main disadvantage is the use of expensive Au metal. Therefore, Deng et al. (2014) have studied the conversion of HMF to FDCDM using cheap cobalt based catalyst (Co_x_O_y_-N@C). However, the catalyst Co_x_O_y_-N@C shows low FDCDM selectivity (38% yield) but produces other products like 44% yield of 2,5-furandicarboxylic acid monomethyl ester (FDCMM) and 12% yield of 5-hydroxymethyl-2-furoic acid methyl ester (HMFM) at 100°C in 1 MPa O_2_ pressure after 12 h. Alternatively, K-OMS-2 was used as the strong oxidant to improve the alcohol group oxidation to aldehyde group and HMFM yield was decreased to 1% but FDCMM was still produced in yield of 41%.

In considering the oxidative esterification of HMF to FDCDM, apart from being able to produce purified FDCA, there is the option to conduct reactions in low amounts of base or base free media. Au has shown to have good catalytic effect and selectivity, but is not cheap. On the other hand, Co is a cheap catalyst with catalytic activity but exhibits low selectivity. Therefore, synthesizing cost-effective bimetallic catalysts consisting mainly of non-noble metal catalyst with high catalytic activity and selectivity should be considered in future research.

## Catalytic Conversion of CMF to FDCA

CMF is a versatile chemical which has similar functionality and HMF and in some aspects, shows better properties than HMF. For example, the direct conversion of cellulosic biomass to CMF can be achieved in a high yield of 71–76% (Mascal and Nikitin, 2008), and is much easier to separate in acidic media due to its hydrophobicity and stability compared to HMF (Mascal, 2015). CMF has been used as a base chemical in the production of biofuels, agrochemical, and pharmaceutical products, such as δ-aminolevulinic acid (Dutta et al., 2015a), ranitidine (Mascal and Dutta, 2011), and prothrin (Chang et al., 2014). Chundury and Szmant (1981) was the first to investigate the use of nitric acid in the oxidation of CMF to FDCA. This was followed by Brasholz et al. (2011) who replicated the same experiment and reported 59% FDCA yield.

Alternatively, CMF can be directly converted into DFF, and then the latter oxidized to diacid chloride of FDCA which is known as furan-2,5-dicarbonyl chloride (FDCC). Vicente et al. (2017) have studied the heterogeneous catalytic oxidation of CMF to DFF by using copper catalysts with the oxidants H_2_O_2_, acetonitrile, oxone, and pyridine N-oxide (PNO). Under microwave irradiation at 160°C in acetonitrile for 5 min, 54% of DFF was formed when the combination of PNO and copper(II) trifluoromethanesulfonate (Cu(OTf)_2_) was used. Mascal and his group was able to oxidized DFF to FDCC with 80% yield in the presence of *tert*-butyl hypochlorite (t-BuOCl). Compared to the FDCA, FDCC is soluble in most of the common solvents whereas, FDCA is only soluble in polar aprotic solvents like DMSO (Dutta et al., 2015b). Also, FDCC functions as a precursor in the production of PEF and other FDCA derived polymers like poly(2,5-furandicarboxylates) (Gomes et al., 2011). Although, CMF can be more easily produced in high yield from biomass than HMF, the overall yield to FDCA is typically lower. However, the conversion of CMF to FDCA is an emerging field, where more development improvements in yield and processability can be achieved.

## Conclusions and Perspectives

Polyethylene terephthalate and polyamides are important polymeric materials vital to the modern economy. Some industries have initiated production of bioplastics or green polymers from bio-chemicals which are derived from lignocellulosic biomass. Among them, bio-chemicals like ethylene glycol (EG) and FDCA have shown successful conversion to bioplastics. FDCA is mainly used to produce biobased polymers, and has also been used in the production of surfactants and polyesters (Jiang et al., 2015; McKenna et al., 2015). Apart from producing bioplastic bottles, FDCA can be used to produce value-added chemicals like succinic acid, adipic acid, and 2,5-dihydroxymethyl tetrahydrofuran and also used in the surfactant industry (Van Es et al., 2013).

Numerous researches have been conducted on the synthesis of FDCA from biomass-derived carbohydrates, are more preferable due to high conversion rate where initially carbohydrates are dehydrated to HMF and then subsequently oxidized to FDCA. This process can be achieved through chemical and biological catalytic conversion as well as electrochemical conversion pathways. However, chemical catalytic methods and high yield of FDCA (Sajid et al., 2018). Among the chemical catalytic methods, heterogeneous catalysts are more suitable due to their easy separation and good recyclability. Mostly catalysts are noble metal catalysts but recently, transitional metal oxide catalysts and non-metal catalysts have also been successfully used for the oxidation of HMF and conversion of biomass to FDCA. In this review, the effect of different oxidants and reaction media on the oxidation of HMF have been discussed.

On the discussion of the oxidants, molecular O_2_ appears most feasible for the oxidation of HMF due to its availability and low cost. However, when O_2_ is used for the oxidation high pressures are generally required and that is the main disadvantage of using O_2_. Instead of O_2_, H_2_O_2_, and *t*-BuOOH have also been used as the oxidant in the oxidation of HMF. However, these oxidants have limited suitability for large scale production due to the explosive nature of peroxides.

The different reaction media was shown to affect reaction pathways, Au-based catalysts oxidized in alkaline media produced HFCA as an intermediate whereas, base-free oxidation proceeds via DFF route. This suggests the aldehyde group oxidation is much quicker than alcohol group oxidation in alkaline media. Also, isotope labeling confirmed that the reaction mechanism of base and base-free environment are similar. Water molecules in the reaction media were found to provide the oxygen source to convert HMF to FDCA and surprisingly not the oxidants. And, the role of molecular oxygen is to substitute electron deposition in the supported metals for the catalyst reimbursement. Even though significant researches have been carried out on the production of FDCA from various biomasses using different catalysts and techniques, there is a scarcity of market share for FDCA in the polymer market. Economically, HMF has similar or higher price than FDCA which raising the question of producing FDCA from high cost feedstocks like HMF. Therefore, extensive research has focused on the aspect of deriving commercial benefit synthesizing FDCA from cheaper feedstocks. Based on the information discussed in this review paper, the following directions are suggested for future research.

- Extensive studies on the effect of reaction media for the catalytic oxidation of HMF to FDCA will help in the selection of effective processes. Alkaline media provides higher yields compared to base-free but is economically and environmentally less feasible. Base-free media is the most preferable, however, generally suffers from low FDCA yield and selectivity in the HMF oxidation to FDCA. Therefore, more studies utilizing base-free catalytic oxidation to improve yield and selectivity of FDCA from HMF are imperative to addressing environmental issues.
- The direct conversion of lignocellulose biomass to FDCA in high yield is required for the competitive value for FDCA as a building block chemical for the production of green products. Furthermore, direct conversion to FDCA should address the problems related to HMF separation from the reaction media. Therefore, designing a catalyst with acid/base sites for carbohydrate dehydration to HMF in a hydrophilic environment and with metal sites for oxidation of HMF to FDCA in hydrophobic media could be effective in the production of FDCA with high yield and selectivity direct from lignocellulose biomass. Where, acid/base sites will adsorb carbohydrates and desorb HMF while metal sites will adsorb HMF and oxidize to FDCA. However, initially glucose has to be released from the lignocellulose biomass and isomerized to enable more efficient conversion to FDCA. Therefore, extensive researches need to be conducted to efficiently enable both release of glucose from the lignocellulose biomass and its isomerization to fructose.
- Various oxidants have been used to convert HMF to FDCA like KMnO_4_, H_2_O_2_, and O_2_. Among them O_2_ has shown the ability to produce high yields of FDCA, however the major drawback is the high pressure usually required to complete the reaction. Therefore, finding a catalyst which can be used at low pressure to efficiently oxidize HMF to FDCA will provide much needed process benefit in terms of reduced energy.
- Various research has been conducted in the improvement of multifunctional groups and acid-base properties of ionic liquids and deep eutectic solvents which assist the catalytic reaction for the production of FDCA. To achieve the goal of direct conversion of lignocellulosic biomass to FDCA it will be advantageous using a solvent with strong catalytic activity. However, the major problem of using ionic liquids and deep eutectic solvents is their environmental impact. Future research should aim to address the environmental issue by developing these solvents from biomass.
- Extensive evaluation of the chemical kinetics and mechanisms for the conversion of HMF to FDCA and sugars to FDCA are required to allow more effective design of new catalysts with high catalytic activity, recyclable and that are economical for the direct conversion of lignocellulose biomass to FDCA.
- The enzymatic HMF conversion to FDCA should be further explored as it is achieved under mild conditions. McKenna et al. (2015) have demonstrated a mix of enzymes (galactose oxidase M3-5 and aldehyde oxidase PaoABC) in a tandem cascade reaction that could be used to convert HMF to FDCA. Enzymatic polymerization is an immerging field which challenges the conventional polymerization techniques due to the use of renewable, non-toxic catalysts and mild reaction conditions (Jiang et al., 2015). Therefore, synthesis of polyesters via enzymatic polymerization of biobased monomers have emerged and biobased dimethyl 2,5-furandicarboxylate (DMFDCA) has been used to produce FDCA. Due to the sustainability and mild reaction conditions, further studies should focus on the production of FDCA and derivative polymers using enzymatic catalysts.
- CMF has similar chemical functionally as HMF. However, it is more versatile in some aspects than HMF due to its ability to directly couple with organometallics, and so a tendency toward nucleophilic substitution and high dissolving power. Therefore, conversion of CMF to FDCA is an emerging field which need extensive study so that an industrial application can be realized with lignocellulosic biomass as the feedstock.
- Finding other higher value applications of FDCA would help to both grow market demand and sustain further research and development. Polyesters and polyamides are important polymer applications that derive greater value than plastic packaging and also represent sustainable market size.

When considering the large-scale production of FDCA, the process will need to be designed to address all technical, economic and environmental challenges. Additionally, energy implications associated with processing low feed loadings which are generally necessary to achieve high FDCA yield will also need to be addressed. This will require interdisciplinary research in material engineering, chemical engineering, and process design.

## Author Contributions

AD, LA, LM, DR, JB, and WD drafting the manuscript. JB and WD coordinating and supervising. LA, LM, DR, JB, and WD supervision and editing. AD writing the original draft. All authors contributed to the article and approved the submitted version.

## Conflict of Interest

The authors declare that the research was conducted in the absence of any commercial or financial relationships that could be construed as a potential conflict of interest.
